# Electromechanical Impedance-Based Hybrid Physical Features and Data-Driven Framework for Simulated Damage Identification and Prediction of Composites in Noisy Environments

**DOI:** 10.3390/polym18161995

**Published:** 2026-08-16

**Authors:** Jianguo Ma, Longlei Dong

**Affiliations:** State Key Laboratory for Strength and Vibration of Mechanical Structures, Shaanxi Key Laboratory of Environment and Control for Flight Vehicle, School of Aerospace Engineering, Xi’an Jiaotong University, Xi’an 710049, China; jianguo.ma@stu.xjtu.edu.cn

**Keywords:** electromechanical impedance, physical features extraction, fast Fourier transform smoothing filter, aerospace composites, noisy environments

## Abstract

Data-driven models are transforming structural health monitoring (SHM) for composites. However, excessive sensor costs and scarce, noise-contaminated data hinder model accuracy and generalizability. In this study, an electromechanical impedance (EMI)-based physical features and data-driven framework for high-precision damage assessment under conditions with noise and limited data is proposed. An experimental system that incorporates random noise to simulate operational environment noise was used to simulate seven progressive simulated damage states in CFRP laminates. Adaptive low-pass parabolic filtering via a fast Fourier transform smoothing filter (FFT-SF) denoised conductance signals in the frequency domain, increased efficiency over the Hinkley criterion, and significantly suppressed false alarms from sensor drift. Three input variables were selected: the resonant frequency F (reflecting structural stiffness), the resonant amplitude A (reflecting structural damping), and the root mean square deviation (RMSD) index (a statistical measure of spectral deviation). These three variables, two physics-based features and one statistical index, formed the inputs to a three-input artificial neural network (ANN). The fusion model achieved an RMSE of 0.0752 and an R^2^ of 0.9807 on 105 small samples, significantly outperforming a purely data-driven single-input RMSD-ANN (RMSE of 0.1066; R^2^ of 0.9612). Critically, Shapley Additive Explanation (SHAP) analysis revealed that the physical features significantly enhance model interpretability and predictive reliability, maintaining >98% simulated damage identification accuracy with extremely limited real data. This provides a cost-effective, high-precision, and scalable paradigm for aerospace composite SHM.

## 1. Introduction

The development of data-driven models is crucial for automated damage detection in structural health monitoring (SHM) [1]. However, the process of establishing data-driven models is subject to checks and balances between the quality of the original monitoring data and the performance of the model [2]. The quality of raw monitoring data is the basis of model performance, which is both mutually constrained and deeply coupled with the accuracy and generalization ability of the model [3,4]. An increase in model accuracy and generalization ability often requires a significant increase in the amount of raw monitoring data or an increase in monitoring quality [5]. This is extremely challenging for practical applications in SHM. The cost of monitoring data in the aerospace field is extremely high, and the quality of the collected data is disturbed by noise [6]. This challenge can be specifically reflected in monitoring the health of aerospace composites in noisy environments [7]. In today’s commercial aerospace field, the reuse of spacecraft can greatly reduce costs [8]. This also puts forward requirements for monitoring the material durability of spacecraft, and damage monitoring is the most important part.

Many SHM methods have been applied to damage monitoring, such as acoustic emission [9], ultrasonic guided waves [10], and fiber optic Bragg (FBG) sensors [11]. Electromechanical impedance (EMI) technology is among the most promising SHM technologies available, with the advantage of being able to quantitatively measure damage with a lightweight, low-cost PZT piezoelectric sensor [12]. In EMI technology, PZT sensors can be used as sensors as well as actuators; on the basis of the direct and reverse piezoelectric effects of piezoelectric materials, the changes in the mechanical impedance of the coupled structure can be detected and reflected by the changes in the electrical impedance or electrical conductance of the PZT sensor [12]. One of the common challenges in damage monitoring is monitoring data drift in sensors caused by external vibration interference and noise [13], which can lead to false positives. This is also evident in EMI technology, where aerospace composites commonly encounter this challenge [14].

Research on the EMI monitoring of composites under noise interference can be traced back to the work of Baptista et al. [14], who effectively resisted the effects of noise interference in EMI damage monitoring using the Hinkley criterion and cross-correlation square deviation (CCSD) [15]. Moreover, a new damage localization imaging algorithm based on EMI technology was developed for further multidamage recognition problems [16], which is still very effective under noise interference. On the basis of the research ideas of Baptista et al. [17], this study used a more advanced anti-noise interference method based on EMI technology combined with data-driven methods. In current research, the following shortcomings and challenges exist: (1) Although signal processing technologies such as the Hinkley criterion and CCSD indicators can effectively reduce the impact of noise interference, false alarms still occur in specific damage identification, so EMI technology that integrates more effective denoising algorithms must be developed. (2) At present, traditional quantitative indicators such as the CCSD, correlation coefficient deviation metric (CCDM) [14] or root-mean-square deviation (RMSD) [12] cannot achieve the early prediction of damage types, which indicates that the real-time early warning needs of the SHM field cannot be met, and data-driven methods have become effective means to solve this problem.

In terms of damage monitoring, many scholars have used EMI technology combined with machine learning or deep learning for automated quantification and prediction purposes in recent years [18]. For example, Meher and Sunny [19] used artificial neural network (ANN) methods to quantify the internal delamination damage to composite lap joints. Ai and Yang [20] used transfer learning methods to automatically identify damage to plate rubber bearings. Deng et al. [21] used EMI technology to integrate deep learning methods to automatically evaluate the durability of the CFRP–concrete interface. In these studies, the accuracy and generalization ability of data-driven methods are excellent, but they are based on a large amount of monitoring data. In dealing with the harsh service environment of aerospace applications, a large amount of monitoring data is unrealistic. In the case of a small data volume, data augmentation methods were used in the studies of Ai and Yang [20] and Deng et al. [21] to increase the data volume, and the resulting false data were used to increase model complexity and prevent overfitting. However, this increases the computational effort of the model, and the data-enhanced false values are not of the highest data quality. Therefore, establishing an excellent data-driven model based on a small amount of real monitoring data has become a key challenge in damage monitoring.

While the individual components discussed above, EMI-based monitoring, signal denoising, and machine learning-based damage classification, have each been explored in the literature, existing studies have not addressed the specific challenge of achieving high-accuracy quantitative damage identification under the combined constraints of strong noise interference and severely limited training data. Notably, the Hinkley criterion and CCSD methods [14,15] provide effective noise suppression but can still produce false alarms and do not extract the underlying physical resonance parameters. Approaches combining EMI features with machine learning [19] typically rely on large training datasets or employ data augmentation to compensate for data scarcity [20,21], which introduces synthetic samples of uncertain fidelity. Moreover, these methods often use statistical indices such as RMSD as the sole input feature, which, as we demonstrate in this work, lack sensitivity to frequency shifts and cannot fully decouple certain simulated damage states. The present study bridges these gaps by proposing an integrated framework that (i) adapts the FFT-SF method specifically for EMI frequency-domain signals to preserve resonance peak fidelity while suppressing noise, (ii) extracts dual physics-based resonance features (frequency F and amplitude A) that capture stiffness and damping changes directly, and (iii) fuses these physical features with the conventional RMSD index in a compact ANN model that achieves high accuracy and interpretability with only 105 real measurements, without recourse to synthetic data augmentation.

To overcome this challenge, methods that combine physical knowledge with data-driven approaches have gained increasing attention [22,23,24]. These methods can be broadly understood in terms of how physical information is incorporated. Physics-informed neural networks [22,23,24] embed governing physical equations as constraints within the model training process, ensuring predictions remain consistent with known principles. At the other end, domain knowledge is used to engineer physically meaningful features from raw sensor data, which are then supplied as inputs to conventional machine learning models. In both paradigms, the guiding principle is the same: physical knowledge compensates for data scarcity and improves model robustness.

Despite these advances, a clear research gap remains in the EMI-based SHM literature. This gap can be articulated along three dimensions. First, regarding noise handling: existing methods such as the Hinkley criterion and CCSD [14,15] stabilize damage indices against noise but do not reconstruct the underlying resonance signature of the conductance spectrum. Without spectral reconstruction, the physically meaningful resonance parameters, frequency F and amplitude A, cannot be reliably extracted under noise interference. Second, regarding feature representation: EMI-ML studies [19,20,21] predominantly employ statistical indices (e.g., RMSD, correlation coefficients) as model inputs. While convenient, these indices collapse the entire spectrum into scalar values and discard the stiffness and damping information physically encoded in the resonance peaks. Third, regarding data requirements: prior data-driven EMI studies typically rely on large training datasets or employ synthetic data augmentation [20,21] to achieve acceptable accuracy, which is impractical for aerospace applications where labeled damage data are scarce and expensive to acquire. To date, no existing study has simultaneously addressed all three challenges: robust noise suppression with resonance preservation, physics-based feature extraction and fusion, and high-accuracy damage identification under small-sample constraints, within a single integrated framework. This three-dimensional gap motivates the present work.

Therefore, this study proposes an EMI-based physical features and data-driven framework for simulated damage identification and prediction of carbon fiber-reinforced plastic (CFRP) composite beams in noisy environments. The original conductance signal is denoised by the efficient fast Fourier transform smoothing filter (FFT-SF) method to extract the physical features of the conductance signal under noise interference (frequency F and amplitude A corresponding to the resonant peak of the conductance signal after denoising). A pure data-driven ANN model after denoising is established by using the RMSD quantitative index, and a multidimensional input ANN model combining hybrid physical features is established by combining the extracted dual-parameter parameters of the resonance peak to realize simulated damage identification and prediction with high precision and low data volume requirements. A comparative summary of representative EMI-based SHM studies, situating the present work within the existing literature landscape, is provided in Appendix A Table A5. This table highlights the key methodological differences across studies in terms of feature types, noise handling capability, data requirements, and interpretability. The main contributions of this work are as follows:

(1) An EMI-specific adaptation of the FFT-SF denoising method is developed, which operates directly on frequency-domain conductance spectra. Unlike existing time-domain FFT-SF applications, this adaptation employs IFFT-to-time-domain filtering followed by FFT reconstruction, enabling effective noise suppression while preserving the physical fidelity of resonant peaks—a capability that previous EMI denoising techniques (e.g., the Hinkley criterion [14]) do not provide, as they focus on damage index stabilization rather than resonance signature recovery.
(2) A dual physics-based feature extraction strategy is proposed, which captures the resonance frequency F and amplitude A from FFT-SF denoised spectra. These features directly encode the stiffness and damping changes of the coupled PZT-CFRP system. We demonstrate that these physics-based features, when combined with the statistical RMSD index, resolve the damage-state decoupling problem that purely statistical metrics cannot address.
(3) A hybrid physical features and data-driven framework is established, integrating the RMSD index with the dual resonance features in a compact three-input ANN. While the principle of fusing physically meaningful features with data-driven models is established in the broader literature, the specific contribution here lies in the EMI-tailored combination: the FFT-SF spectral reconstruction enables reliable extraction of resonance features under noise, a capability that prior EMI denoising methods do not provide, and the integration of these features with RMSD in a lightweight ANN is shown to achieve high accuracy and SHAP-based interpretability under small-sample conditions, addressing the combined noise and data scarcity challenges specific to EMI-based SHM.

## 2. Electromechanical Impedance Method

**Figure 1:**
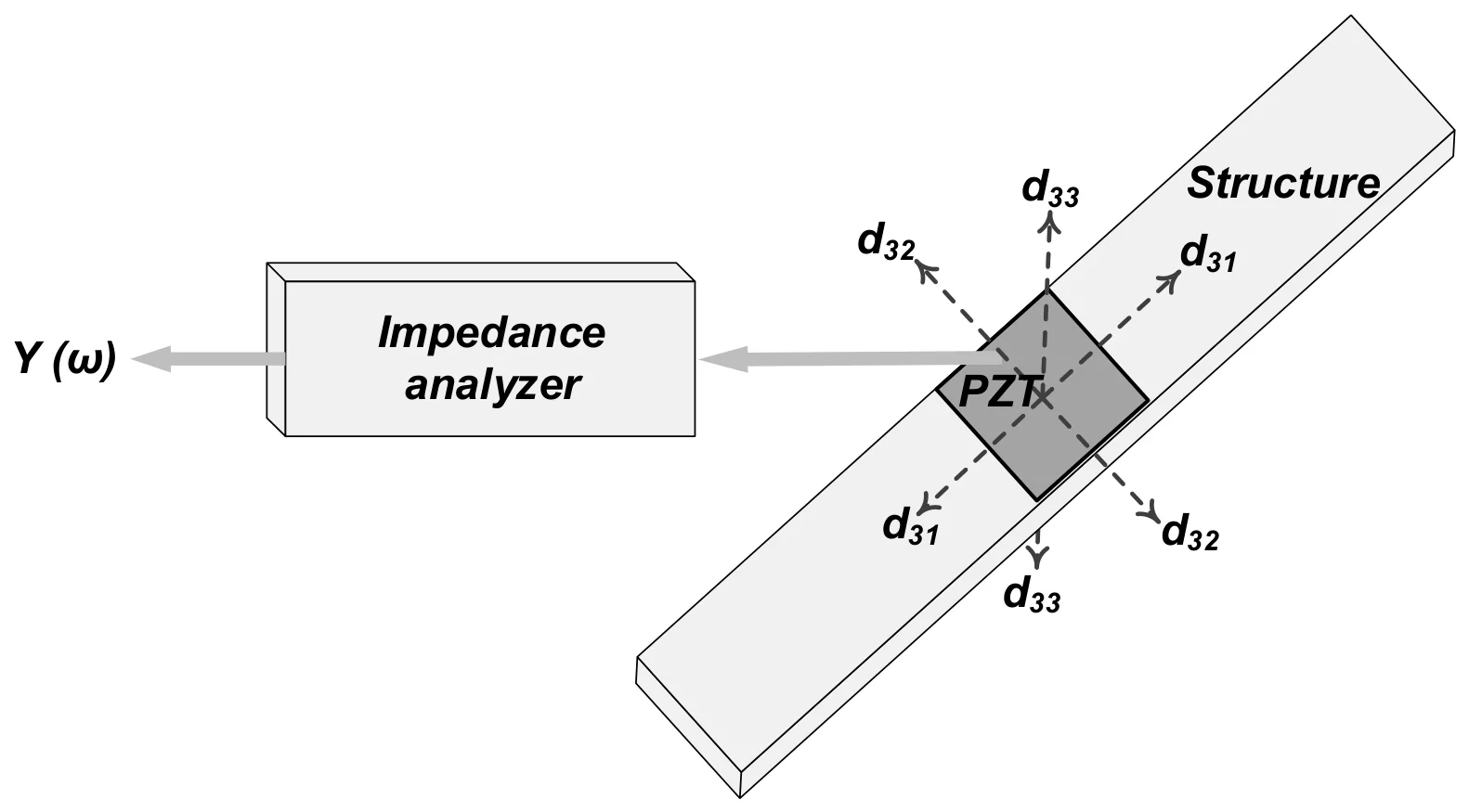
PZT sensor directly bonded to the main structure.

Electromechanical impedance (EMI) technology is an SHM method that utilizes the effect of electromechanical coupling between piezoelectric lead zirconate titanate (PZT) sensors and the main structure. There are several common electromechanical coupling configurations [25,26,27], the most common of which is used in this study to directly bond the PZT sensor to the main structure, as shown in Figure 1. In EMI measurements, the PZT sensor is electromechanically coupled to the main structure and records the electrical impedance as it vibrates within a specified frequency range. The amplitude of the sensor’s vibration can be adjusted by changing the applied AC voltage. Measuring the captured sensor impedance can reflect the change in the mechanical impedance of the sensor, thereby indirectly reflecting the mechanical impedance of the main structure [28].

The PZT sensor monitors the mechanical properties of the main structure through its own direct and reverse piezoelectric effects, and the basic constitutive relationship regarding the direct and reverse piezoelectric effects of piezoelectric materials is as follows [29]:

(1) $$D=d.T+\varepsilon^{T}.E$$

(2) $$S=s^{E}.T+d^{t}.E$$

where S represents the mechanical strain component, D represents the electrical displacement component, T represents the mechanical stress component, $s^{E}$ represents the complex elastic compliance coefficient when the electric field strength is zero or constant, $\varepsilon^{T}$ represents the complex dielectric constant when the stress is zero or constant, $d^{t}$ and d represent the piezoelectric strain constant, and E represents the electric field strength component. In Equations (1) and (2), the electrical and mechanical properties of piezoelectric materials are nonisotropic. Therefore, an electromechanical coupling model must be established to correlate the mechanical properties and electrical impedance of PZT sensors [30].

With respect to the electromechanical coupling model, a simple spring–mass–damper model driven by an actuator was first proposed by Liang et al. [31] to simulate the one-dimensional electromechanical coupling between a PZT sensor and its main structure. The electromechanical admittance $Y\left(\omega \right)$ of a PZT sensor can be expressed as follows:

(3) $$Y\left(\omega \right)=i\omega \frac{A_{P}}{h_{A}}\left[{\bar{\varepsilon }}_{33}^{\sigma }-d_{31}^{2}{\bar{Y}}_{33}^{E}+\frac{Z_{A}{\left(\omega \right)d}_{31}^{2}{\bar{Y}}_{33}^{E}}{Z_{S}\left(\omega \right)+Z_{A}\left(\omega \right)}\frac{\tan\left(kl_{A}\right)}{kl_{A}}\right]$$

where ω is the excitation frequency and $l_{A}$, $A_{P}$, and $h_{A}$ are the diameter, surface area, and thickness of the PZT patch, respectively. ${\bar{\varepsilon }}_{33}^{\sigma }=\varepsilon_{33}^{\sigma }\left(1-\delta i\right)$ is the complex dielectric constant of the PZT patch at zero stress, δ is the dielectric loss factor, ${\bar{Y}}_{33}^{E}=Y_{33}^{E}\left(1+\eta i\right)$ is the complex elastic modulus of the PZT patch at zero electric field, η is the mechanical loss factor, $d_{31}$ is the piezoelectric constant of the PZT patch, and k is the wave number. The mechanical impedance $Z_{A}\left(\omega \right)$ of the PZT patch and the impedance $Z_{S}\left(\omega \right)$ of the main structure can be expressed as follows:

(4) $$Z_{A}\left(\omega \right)=\frac{{\bar{Y}}_{33}^{E}h_{A}}{\omega i}\frac{kl_{A}}{\tan\left(kl_{A}\right)}$$

(5) $$Z_{S}\left(\omega \right)=\frac{\bar{\sigma }A_{P}}{i\omega \bar{x}}$$

where $\bar{\sigma }$ and $\bar{x}$ represent the amplitudes of the periodic stress and displacement at the PZT-structure surface, respectively. Equation (3) shows that the change in the mechanical impedance of the structure caused by local damage causes a change in the electromechanical admittance of the PZT sensor. In EMI technology, the most reasonable swept-frequency range and electromechanical admittance characteristic are applied to detect structural damage to maximize the sensitivity of PZT sensors [25]. Moreover, as described later in this study, environmental noise corrupts the electromechanical admittance characteristic, thereby affecting the detection of structural damage [14].

### Physical Interpretation of the Resonance Features

The physical basis for using the resonant frequency F and amplitude A as structural state indicators follows directly from the EMI admittance model (Equation (3)). The conductance G(ω), defined as the real part of the electromechanical admittance Y(ω), reaches a local maximum when the combined impedance of the PZT sensor and the host structure is minimized. This condition defines the resonance peak observed in the measured conductance spectrum.

The frequency at which this resonance occurs is governed primarily by the effective stiffness and mass of the coupled PZT-structure system. When the structure undergoes stiffness degradation, for instance, due to delamination or ply separation in a CFRP laminate, the local effective stiffness decreases, causing the resonance frequency to shift downward. Thus, a decreasing trend in F reflects a reduction in structural stiffness.

The amplitude of the conductance at the resonance peak is governed primarily by the effective damping of the coupled system, which includes both the intrinsic mechanical losses of the PZT sensor and the energy dissipation characteristics of the host structure. Damage mechanisms that increase internal friction, such as matrix cracking or interfacial debonding, increase the effective damping and consequently reduce the peak conductance amplitude. Thus, a decreasing trend in A reflects an increase in structural damping.

These relationships establish F and A as physically interpretable indicators of stiffness- and damping-dominated structural changes, respectively. This physical foundation justifies their use as features in the data-driven damage identification framework and provides the basis for interpreting the SHAP analysis results presented in Section 5.2.3.

## 3. Experimental Setup

To evaluate the effect of the noise environment on EMI technology in simulated damage identification, several damage experiments were carried out using a carbon fiber-reinforced plastic (CFRP) composite beam with dimensions of 545 mm × 40 mm × 3 mm. The beams were fabricated from a [0/90/0/90/0/90] cross-ply laminate, with a fiber volume fraction of approximately 60% as reported by the prepreg manufacturer. The detailed properties of the reinforcement carbon fiber fabric and the epoxy matrix are summarized in Table 1.

**Figure 2:**
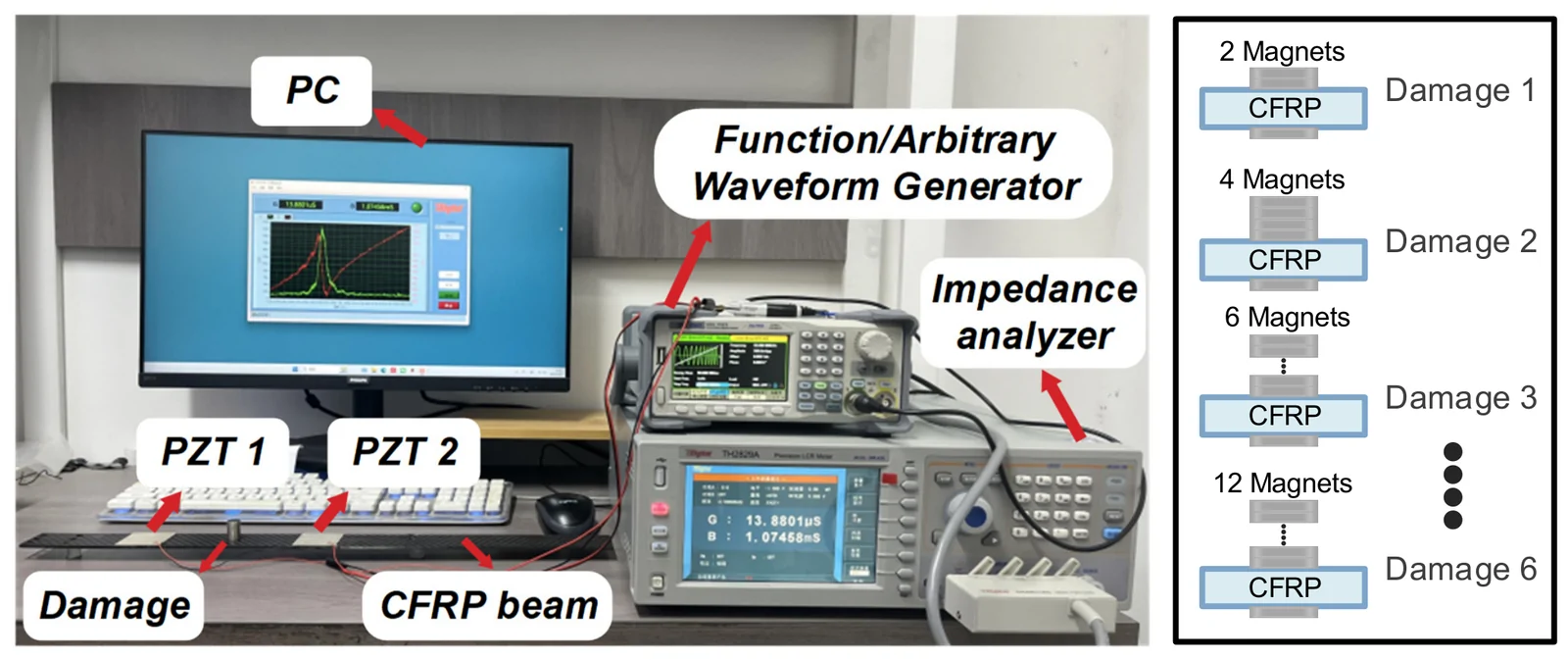
Overall device used in the CFRP beam damage experiment in a noisy environment.

In this study, damage to CFRP beams was monitored using PZT sensors, and square sheets made of the PZT-5 type (Zibo Yuhai Electronic Ceramics Co., Ltd., Zibo, China) with dimensions of 40 mm × 40 mm × 1 mm were selected; the material parameters are shown in Table 2. The PZT sensor was tightly bonded to the upper end of the CFRP beams by cyanoacrylate-based glue, as shown in Figure 2. Two PZT sensors were used in the experiments, one of which was used as an actuator to generate ambient noise, denoted PZT 1. The other was used for EMI measurements and is denoted PZT 2. PZT 1 was installed 85 mm from the beam’s boundary, and PZT 2 was installed in the center of the beam. The damage was set at the geometric center of the two PZT sensors of the beams. To create repeatable and controllable “damage” states for methodological validation, we adopted a magnet-based approach inspired by the work of Singh et al. [32], who demonstrated that local mass and stiffness variations can effectively simulate property changes in composite structures for SHM testing. Specifically, we used NdFeB magnets attached to the beam surface to introduce progressive local mass and stiffness perturbations that mimic the effects of structural degradation on the electromechanical impedance response. A total of 13 pieces of NdFeB strong magnets with a diameter of 12 mm and a thickness of 2 mm were used, and one of them was placed on the lower surface of the damaged location. The remaining magnets were divided into 6 groups (2 pieces each) and adsorbed on the upper surface of the damaged location, as shown in Figure 2. The 7 simulated damage states of the beams can be obtained: Health, Simulated Damage-1, Simulated Damage-2, Simulated Damage-3, Simulated Damage-4, Simulated Damage-5, and Simulated Damage-6.

It is important to clarify the physical relationship between the magnet-induced structural changes and actual composite damage, as this bears directly on the transferability of the proposed framework. The magnets introduce two simultaneous perturbations: a localized mass loading and a compressive stress field at the attachment point. The mass loading increases the local effective density of the beam, while the compressive stress modifies the local contact stiffness between plies. These perturbations produce changes in the electromechanical impedance signature, specifically, a leftward shift in the resonant frequency F and a decrease in the peak amplitude A, that are mechanistically analogous to certain effects of real damage. Delamination, for instance, reduces local bending stiffness by decoupling adjacent plies, which would similarly shift the resonance frequency leftward. Matrix cracking increases internal friction and energy dissipation, which would manifest as increased damping and reduced peak amplitude. However, we acknowledge three important differences: (a) real delamination involves nonlinear contact dynamics at the delaminated interfaces that may produce amplitude-dependent resonance shifts not captured by the linear magnet-induced perturbation; (b) distributed matrix cracking affects a larger volume of material and may influence multiple resonance modes simultaneously, whereas magnet loading is localized; and (c) fiber breakage introduces abrupt, irreversible stiffness discontinuities that may produce qualitatively different spectral features from the smooth trends observed under progressive magnet loading. Despite these differences, the core methodology- FFT-SF spectral reconstruction, physics-based resonance feature extraction, and ANN-based state mapping- is agnostic to the specific perturbation mechanism and should remain applicable provided that the induced EMI signature changes are measurable. Validation on specimens with authentic impact-induced or fatigue-induced damage is therefore the essential next step to quantify the framework’s sensitivity and specificity to these more complex, realistic damage signatures.

We emphasize that the magnet-based approach employed herein simulates reversible local mass and stiffness perturbations rather than the irreversible microstructural damage mechanisms characteristic of CFRP composites, such as delamination, matrix cracking, and fiber breakage. Delamination involves the separation of adjacent plies with complex interfacial contact dynamics; matrix cracking creates distributed networks of micro-cracks that increase internal friction; and fiber breakage introduces abrupt, localized stiffness discontinuities. These real damage mechanisms involve coupled, progressive degradation processes that may produce EMI signatures differing quantitatively or qualitatively from the smooth, monotonic trends induced by magnet loading. Consequently, the term simulated damage is used throughout this manuscript to accurately reflect the nature of the structural state changes investigated.

To ensure that the EMI test was isolated from the environment, the CFRP beams were padded with rubber blocks, and all the EMI measurements were performed at a constant room temperature (approximately 25 °C) to mitigate the impact of temperature changes on the EMI measurements. Moreover, subsequent EMI analyses used the real part of the electrical admittance, namely conductance [28]. More recent investigations have demonstrated that conductance spectra can still be affected by significant temperature changes [33]. Therefore, all measurements in this study were conducted under controlled, constant-temperature conditions to minimize thermal effects. The incorporation of active temperature compensation techniques will be addressed in future work.

Although the present experiments were conducted under constant-temperature conditions to isolate the effects of noise, practical SHM applications inevitably involve ambient temperature variations that can independently influence the EMI signature. Temperature changes affect the conductance spectrum through multiple pathways: the elastic modulus of both the PZT material and the CFRP host structure is temperature-dependent, causing shifts in the structural resonance frequencies; thermal expansion alters the PZT-structure bonding conditions and the local stress state; and the piezoelectric and dielectric constants of the PZT sensor vary with temperature. These thermally induced changes can produce shifts in F and A that are comparable in magnitude to those caused by incipient structural degradation, potentially confounding damage identification if left uncompensated. However, the FFT-SF denoising component of the proposed framework would remain effective under varying temperature, as it operates on the spectral morphology rather than on absolute frequency or amplitude values. The ANN model, trained on data acquired at a single temperature, would likely misclassify temperature-induced feature shifts as structural state changes. Addressing this limitation requires either incorporating temperature as an additional input feature to the ANN, or applying temperature compensation techniques, such as baseline correction using the susceptance signature (the imaginary part of the admittance, which is more temperature-sensitive than conductance and can serve as a temperature indicator), or empirical correction factors derived from temperature calibration experiments. These strategies represent important directions for extending the framework to field applications where temperature control is not feasible.

**Figure 3:**
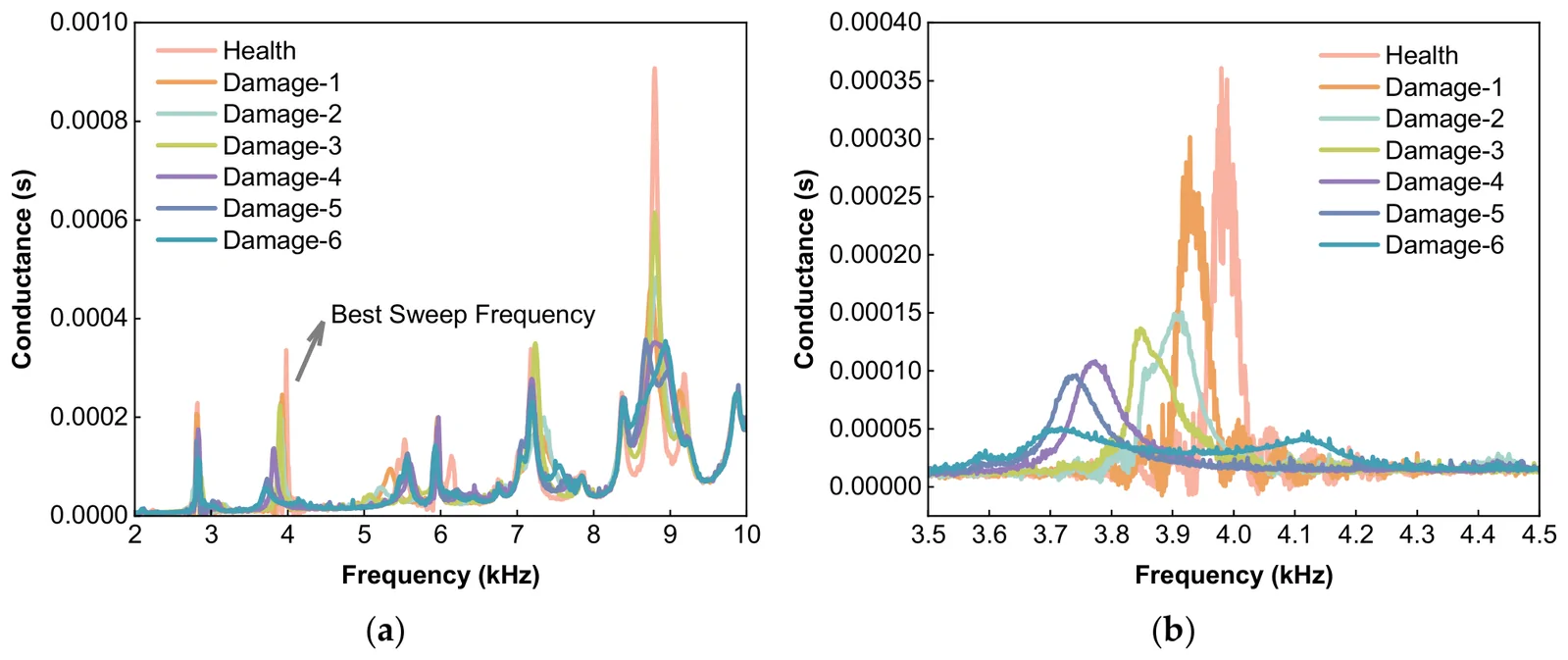
(**a**) Conductance measurement in the initial sweep range of 2 kHz–10 kHz. (**b**) Conductance measurement at 3.5 kHz–4.5 kHz via fine sweep.

In this study, a precision impedance analyzer (TH2829A; Changzhou Tonghui Electronics Co., Ltd., Changzhou, China) was used to perform EMI testing on the CFRP beams. The conductance signal was acquired from a 1 V excitation voltage signal acting on PZT2, and the conductance signal for each EMI measurement consisted of 801 sampling points. According to previous studies [34], the most critical aspect of EMI technology is determining the optimal sweep range, which is similar to relying on the prior knowledge of experts for initial diagnosis. Not finding the optimal sweep range greatly affects the sensitivity of the PZT sensor, and in severe cases, false alarms or discrimination failures can occur. In this study, the EMI sweep range in composite materials was determined at an initial range of 2 kHz–10 kHz with a resolution of 10 Hz, and the EMI test results of damage are shown in Figure 3a. As shown in the Figure 3b, in the range of 3.5 kHz–4.5 kHz with a resolution of 1.25 Hz, the frequency F of the peak shifts to the left with increasing degree of damage (reflecting the change in the stiffness of the main structure). The amplitude A of the peak decreases with increasing damage (reflecting the change in the damping of the main structure) [25]. In this frequency band, the peak has the best trend, whereas the resonant peak behaves irregularly in the remaining frequency bands, which means that it is the best frequency band for monitoring the damage to the beams. In this study, fine measurements were carried out in the range of 3.5 kHz–4.5 kHz, and the fine measurement results of the changes in preliminary damage are shown in Figure 3b.

In this study, a function/arbitrary waveform generator (SDG 1032X, SIGLENT, Shenzhen, China) was used to generate pseudorandom white noise in the frequency band from 0 kHz to 65 kHz and in the amplitude range of 0.2 Vpp–10 Vpp and was applied to the beams via PZT 1. Each sweep time was 50 ms. Therefore, the sweep speed of noise excitation was much higher than that of EMI measurements (Each sweep time = 801 × 9 ms = 7209 ms). The wideband noise up to 65 kHz was employed to simulate a comprehensive and harsh operational noise environment, covering not only the spectral range of the EMI measurement (up to 10 kHz) but also higher-frequency disturbances commonly present in aerospace applications (e.g., engine harmonics, aerodynamic noise). The white noise in this work is generally a random signal that occurs in any range of the EMI spectrum. This is essentially consistent with the setup used in De Castro et al. [14], as this study is an extension of their work. Although the EMI measurements were performed only up to 10 kHz, the out-of-band high-frequency noise can still affect the measurement through the following mechanisms. First, the PZT sensor exhibits a broadband electromechanical response: although its sensitivity is highest near its thickness-mode resonance (typically hundreds of kHz for a 1 mm thick PZT-5 element), it retains non-negligible response at lower frequencies through its compliance-dominated regime. Vibrations induced at higher frequencies by PZT 1 propagate through the beam as guided elastic waves and, through the direct piezoelectric effect, generate a measurable electrical response at PZT 2 across a broad spectrum. This broadband electromechanical coupling means that high-frequency mechanical excitation can contribute to the measured electrical signal even within the 2–10 kHz measurement band. Second, the impedance analyzer’s input stage, while designed with anti-aliasing filters, may experience residual nonlinear effects when subjected to strong out-of-band signals, potentially introducing intermodulation products that fall within the measurement bandwidth. The broadband noise (0–65 kHz) was deliberately chosen to create a conservative worst-case testing condition that challenges both the electromechanical and electronic pathways of the measurement chain, consistent with the testing philosophy adopted by De Castro et al. [14] and Baptista et al. [15,16,17]. By demonstrating effective denoising under these conditions, we establish a baseline of robustness that can inform subsequent testing under more realistic, structured noise spectra.

Moreover, owing to the limitations of the experimental equipment, the maximum amplitude could not be increased to 20 Vpp. Subsequent EMI measurements in the noisy environment gradually applied noise signals of different sizes in each simulated damage state so that each group had 15 conductance results in the simulated damage state, with amplitudes of 0.0 Vpp, 0.2 Vpp, 0.4 Vpp, 0.8 Vpp, 1.0 Vpp, 1.2 Vpp, 1.4 Vpp, 1.6 Vpp, 1.8 Vpp, 2.0 Vpp, 4.0 Vpp, 6.0 Vpp, 8.0 Vpp and 10.0 Vpp. The corresponding signal-to-noise ratio (SNR), defined as the ratio of the RMS conductance signal at 0.0 Vpp to the RMS noise amplitude in the 3.5–4.5 kHz band, ranged from approximately 28 dB at 0.2 Vpp to approximately −6 dB at 10.0 Vpp, covering conditions from mild interference to severe noise corruption. In this study, the noise excitation was maintained until each EMI measurement was completed, after which it was switched to the next amplitude level. A total of 105 sets of data were collected for all impairment states.

## 4. Signal Processing

### 4.1. Basic Damage Indicator

**Figure 4:**
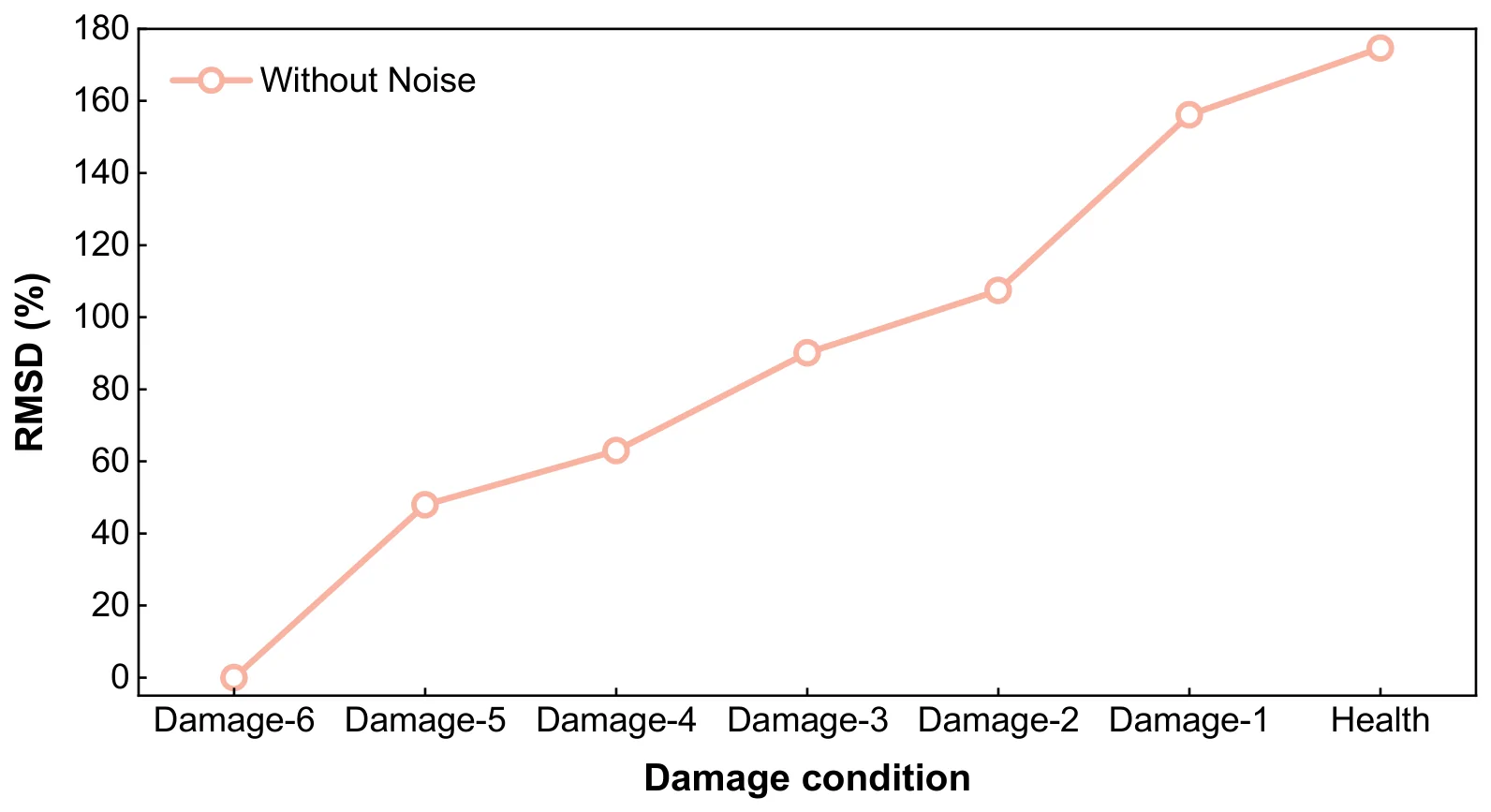
Changes in the RMSD with simulated damage state under the influence of noiselessness.

To further quantify the changes in the conductance signals, the root mean square deviation (RMSD) was used for damage characterization. In this study, the RMSD was used instead of the CCDM index used in the study by De Castro et al. [14], as the RMSD is more effective than the CCDM in characterizing injury [35]. The root mean square deviation (RMSD) is a statistical indicator that reflects the degree of difference between the current state and the reference state [36], and the formula for its calculation is as follows:

(6) $$RMSD\;\left(100\%\right)=\sqrt{\frac{\sum_{i=1}^{N}{\left(G_{i}^{r}-G_{i}^{0}\right)}^{2}}{\sum_{i=1}^{N}{\left(G_{i}^{0}\right)}^{2}}}\times 100\%$$

where $G_{i}^{0}$ and $G_{i}^{r}$ are the i-th conductance characteristics measured in the reference state and the current state, respectively, and N is the number of sampling points for each conductance curve. The larger the RMSD is, the greater the difference between the current state and the reference state. The 105 sets of data measured were characterized by (Simulated Damage-6, 0.0 Vpp) as the baseline. The trend of the RMSD with respect to the simulated damage state under the influence of noiselessness is shown in Figure 4, indicating its effectiveness in damage quantification.

### 4.2. FFT- Smoothing Filter Method

Although the RMSD is an effective means of damage quantification, Campeiro et al. [37] reported that the RMSD indicators are significantly affected by external vibration and noise environments. Many signal processing techniques have been applied to denoising problems for health monitoring [14,15,16]. The Hinkley criterion [14] and cross-correlation square deviation (CCSD) [15] proposed by Baptista et al.’s [17] research group for EMI denoising effectively address the resistance to environmental noise in EMI measurements. They reported that although the Hinkley criterion and CCSD greatly improved the impact of noise on EMI measurements compared with conventional impedance signals combined with damage indicators, they could not completely separate the damage types, and false alarms could still be seen. Therefore, an EMI-based fast Fourier transform smoothing filtering method (FFT-SF) is proposed to solve the overload and distortion problems caused by noise excitation in conductance signals. Moreover, the conductance signals processed by FFT-SF still have obvious physical resonant peak information under noise interference, which provides key support for establishing a hybrid physical features and data-driven combination framework in this study. In the following section, the principle and implementation of FFT-SF are described, namely, the principle of FFT-SF and the improved FFT-SF over previous methods. The dual-parameter extraction of resonant peaks in physical features is shown in Section 4.3, and a discussion of the hybrid physical features and data-driven combination framework is presented in Section 5.2.3.

FFT-SF is an advanced and efficient signal processing technique that usually transforms the time-domain signal into the frequency domain through fast Fourier transform (FFT), applies a low-pass parabolic filter to the frequency domain for denoising, and then returns to the time domain through inverse fast Fourier transform (IFFT) to obtain a smoothed signal. In this technology, the high-frequency component is weakened or removed, and the noise is suppressed, resulting in a smoother signal curve.

**Figure A1:**
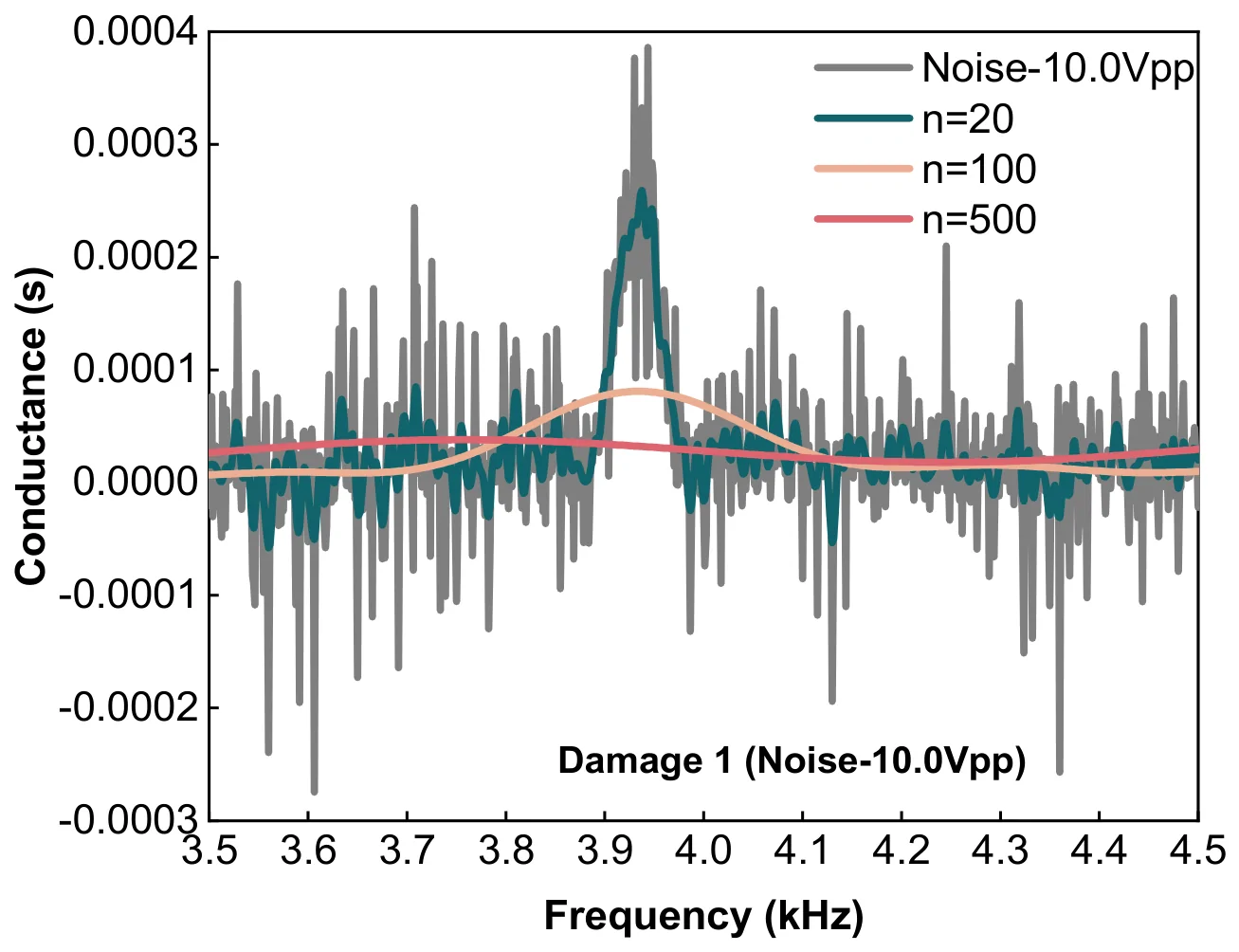
Effect of the smoothing parameter n on the FFT-SF smoothing performance, showing the raw spectrum and smoothed spectra for n = 20 (under-smoothed), n = 100 (optimal), and n = 500 (over-smoothed).

However, in EMI technology, the original measured conductance signal is in the frequency domain; thus, the FFT-SF procedure must be adapted accordingly. First, IFFT is performed on the original frequency-domain conductance spectrum (the real part of the electrical admittance). Since the conductance spectrum is a real-valued function, its inverse Fourier transform yields a Hermitian-symmetric sequence in the reverse time domain. In principle, this sequence is complex-valued; however, because the original frequency-domain data are purely real, the imaginary part of the IFFT result is zero to within numerical precision. The subsequent filtering operation is therefore applied solely to the real part of the IFFT-transformed sequence, which contains the complete information of the original conductance signal. A low-pass parabolic filter is then used to filter the reverse-ordered time-domain signals. The principle is that the Fourier component of the existing time is greater than the cut-off time [38]. The formula for defining the deadline is as follows:

(7) $$t_{cutoff}=\frac{1}{2n∆f}$$

where n is the specified window point and ∆f is the frequency spacing between two adjacent data points (i.e., the horizontal axis of the conductance curve). The larger the n value is, the less the cut-off time and the greater the smoothness. In this study, n was set to 100 for all noise levels after a systematic evaluation of the trade-off between noise suppression and signal fidelity. The selection was guided by two criteria: (1) at the highest noise level (10.0 Vpp), the smoothed spectrum must exhibit a clearly identifiable resonant peak whose frequency and amplitude can be reliably extracted; (2) at the lowest noise level (0.2 Vpp), the smoothing must not cause excessive attenuation or broadening of the peak relative to the noise-free (0.0 Vpp) reference. As illustrated in Appendix A Figure A1, n = 20 fails the first criterion; residual noise obscures the peak shape at high noise levels, while n = 500 fails the second criterion; the peak width is substantially broadened, indicating over-smoothing. The intermediate value n = 100 satisfies both criteria across all noise levels. No noise-level-dependent tuning was required because the parabolic filter adaptively attenuates frequency components above the cutoff frequency defined by Equation (7), providing consistent performance across the tested noise range. The function used to remove the high-frequency component is a parabola, with a maximum value of 1 at time zero and a decrease to zero at the cut-off time [38]. After denoising, the reverse-ordered time-domain signal is transferred back to the frequency domain by FFT to obtain the conductance signal after complete filtering and denoising.

**Figure 5:**
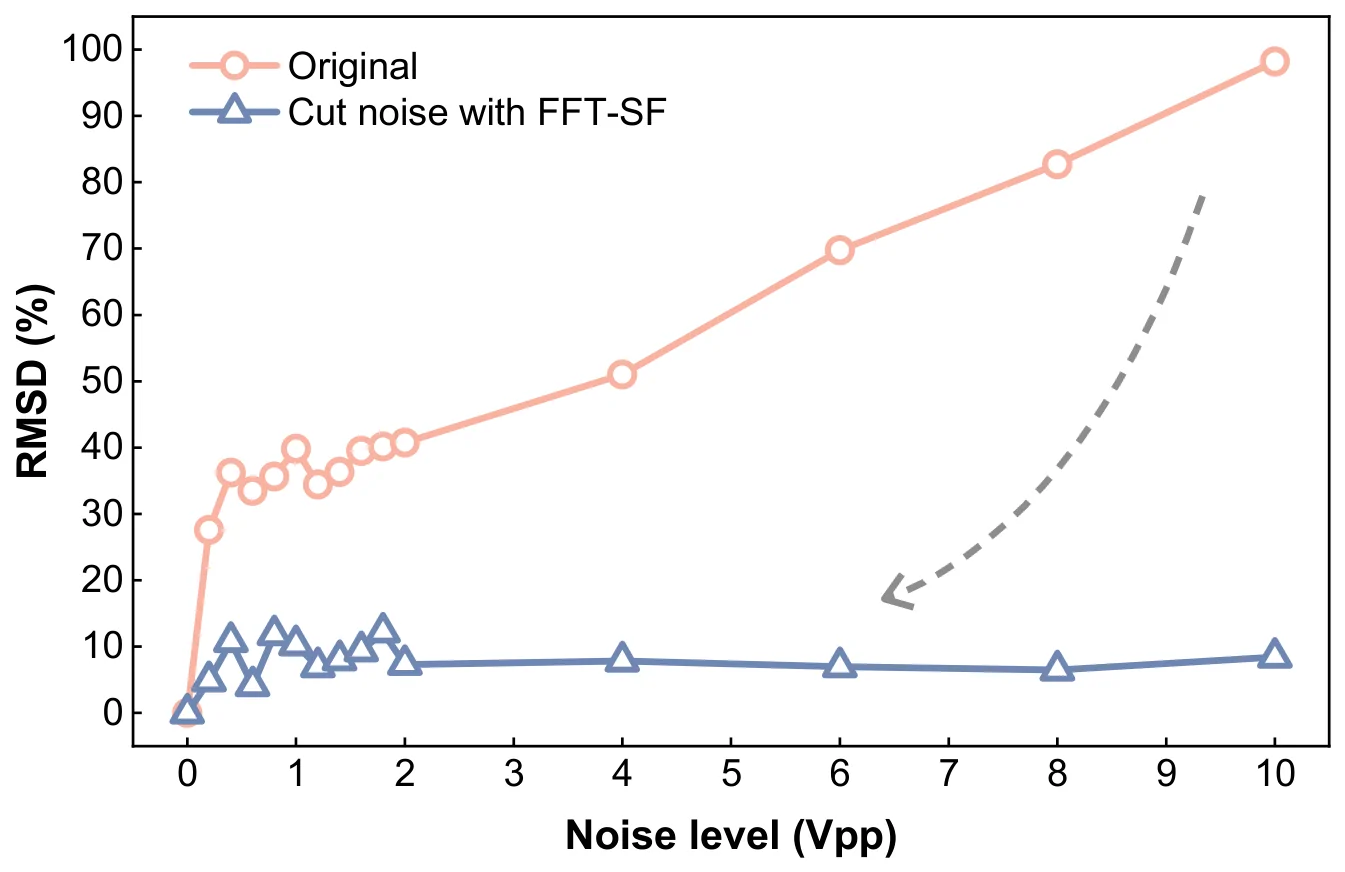
Comparison of Simulated Damage-1 before and after FFT-SF treatment at different noise levels.

**Figure A2:**
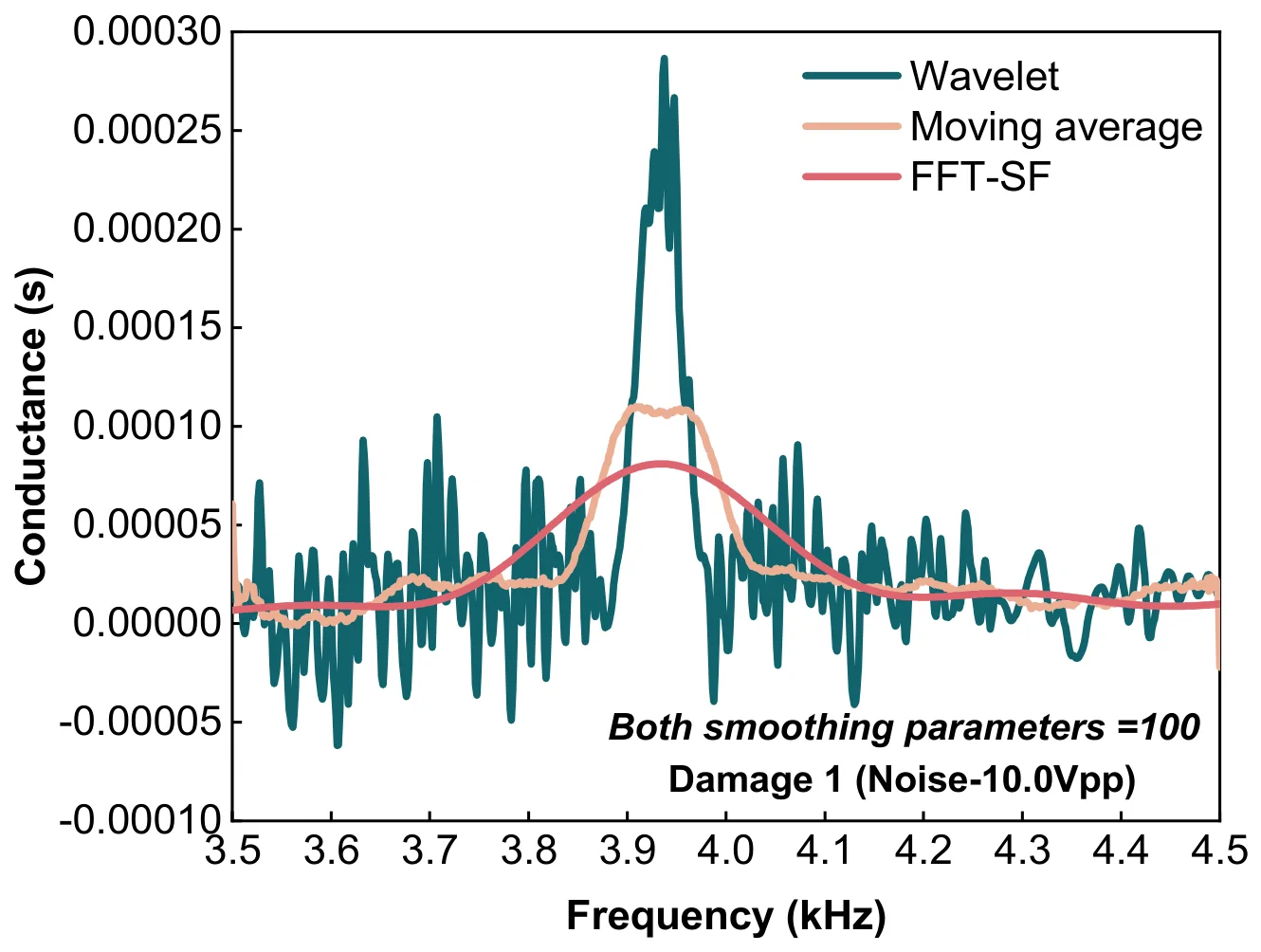
Comparison of smoothing performance among the moving average filter, wavelet denoising (Daubechies db4 wavelet, periodic extension, 100% detail coefficient truncation ratio), and the FFT-SF smoothing method (n = 100) applied to a representative noise-contaminated EMI conductance spectrum.

For the effectiveness of FFT-SF, the RMSD trend for damage at different noise levels is shown here, and the effects before and after the FFT-SF treatment are compared. The conductance curve at 0.0 Vpp was selected as the baseline to ensure consistency with the CCDM treatment studied by De Castro et al. [14], as shown in Figure 5. Here, only the RMSD variation for the Simulated Damage-1 state is displayed; the denoising effects for other states are consistent with Simulated Damage-1, and the regularity can be seen in Figure 9. As shown in Figure 5, the RMSD is greatly reduced after noise removal and is close to zero. This means that the noise effect becomes constant during the damage process and that the noise interference is greatly eliminated. Compared with the Hinkley criterion treatment in De Castro et al. [14], the denoising coefficient (DC) is significantly improved, and the DC calculation formula is as follows:

(8) $$DC\;\left(100\%\right)={[(S}_{o}-S_{c})/S_{o}]\times 100\%$$

where $S_{o}$ represents the area surrounded by the lower part of the damage index curve and the noise level (x-axis) before denoising, and $S_{c}$ represents the area surrounded by the lower part of the damage index curve and the noise level (x-axis) after denoising. The principal advantage of FFT-SF (DC = 87.8%) over index-stabilization methods such as the Hinkley criterion and CCSD lies in a fundamental difference in denoising philosophy. Index-stabilization methods operate on the scalar damage metric: they detect and suppress noise-induced fluctuations in the RMSD or CCDM trend, thereby reducing false alarms, but they do not recover the underlying conductance spectrum from the noise-corrupted measurement. The raw spectrum remains distorted; only the derived index is stabilized. In contrast, FFT-SF performs spectral reconstruction: it removes high-frequency noise components directly from the conductance curve and reconstructs a clean spectrum in which the resonant peak shape, frequency, and amplitude are preserved. This spectral reconstruction capability, which index-stabilization methods do not provide, is the essential prerequisite for extracting the physics-based resonance features F and A. Without a reconstructed spectrum, these features remain inaccessible under noise interference. A comparative analysis with moving average filtering and wavelet denoising is provided in Appendix A, Figure A2.

### 4.3. Physical Resonance Peak Feature Extraction

**Figure 6:**
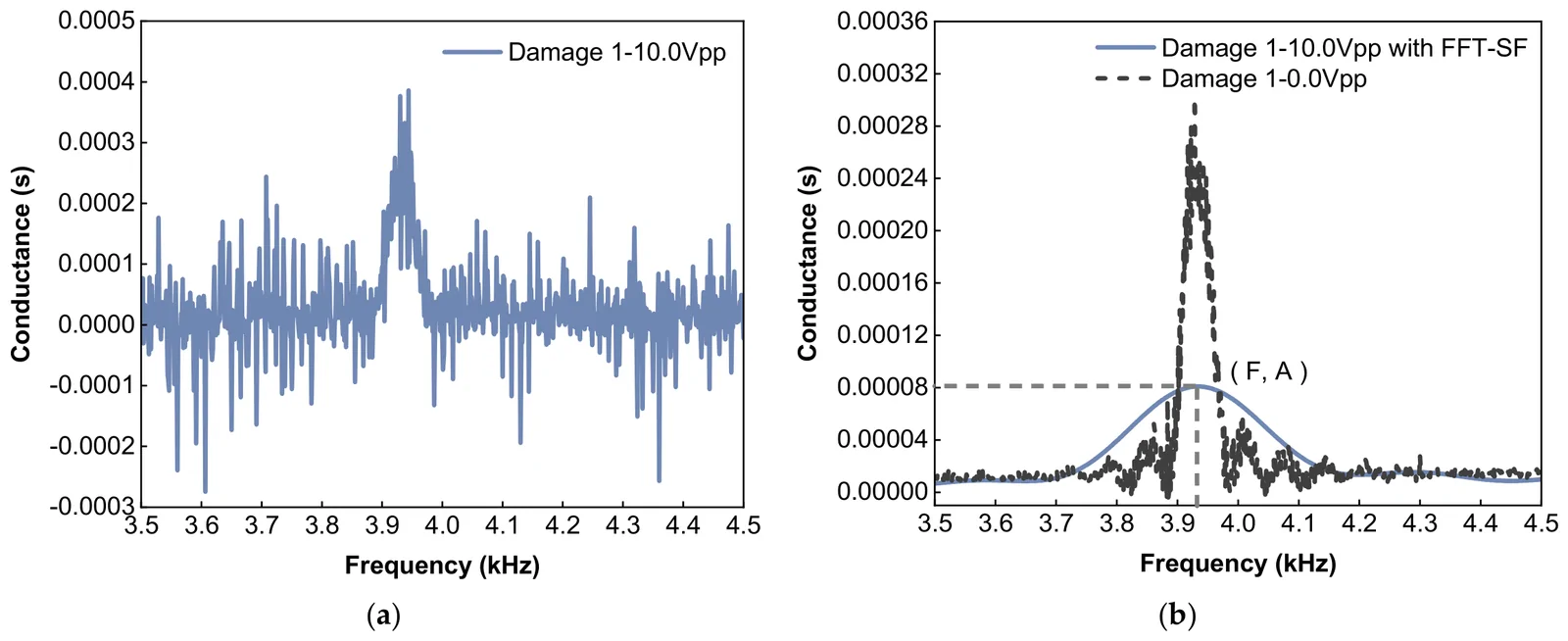
(**a**) Conductance curve for Simulated Damage-1 at a noise level of 10.0 Vpp. (**b**) Extraction of the physical parameters of the resonant peaks from the FFT-SF denoised curve. The dashed line represents the original noise-free (0.0 Vpp) conductance curve, showing the true peak amplitude and frequency that the FFT-SF method effectively recovers.

The conductance curve of Simulated Damage-1 at a noise level of 10.0 Vpp is shown in Figure 6a, which reveals that noise interference with the EMI measurements is extremely obvious. Our previous studies have demonstrated that the frequency and amplitude of the conductance curve represent extremely important physical parameters (frequency refers to stiffness and amplitude refers to damping) for the PZT sensor and the main structure [25]. Ambient noise obscures this information, which is extremely detrimental to damage monitoring. The limitation of traditional RMSD indicators is that they can only establish the trend for the entire frequency band and contain too much useless/misleading information about damage monitoring. However, the resonant peak shift was shown to be a more effective indicator of structural performance changes in the studies of Ma et al. [25] and Zhang et al. [35]. The conductance curve processed by FFT-SF, which shows how to extract the physical parameters of the peaks, is shown in Figure 6b. For reference, the original noise-free (0.0 Vpp) conductance curve is also plotted as a dashed line in Figure 6b, demonstrating that the FFT-SF method recovers both the peak amplitude and frequency with high fidelity.

## 5. Results and Discussion

### 5.1. EMI and Basic Damage Indicator Variation

**Figure 7:**
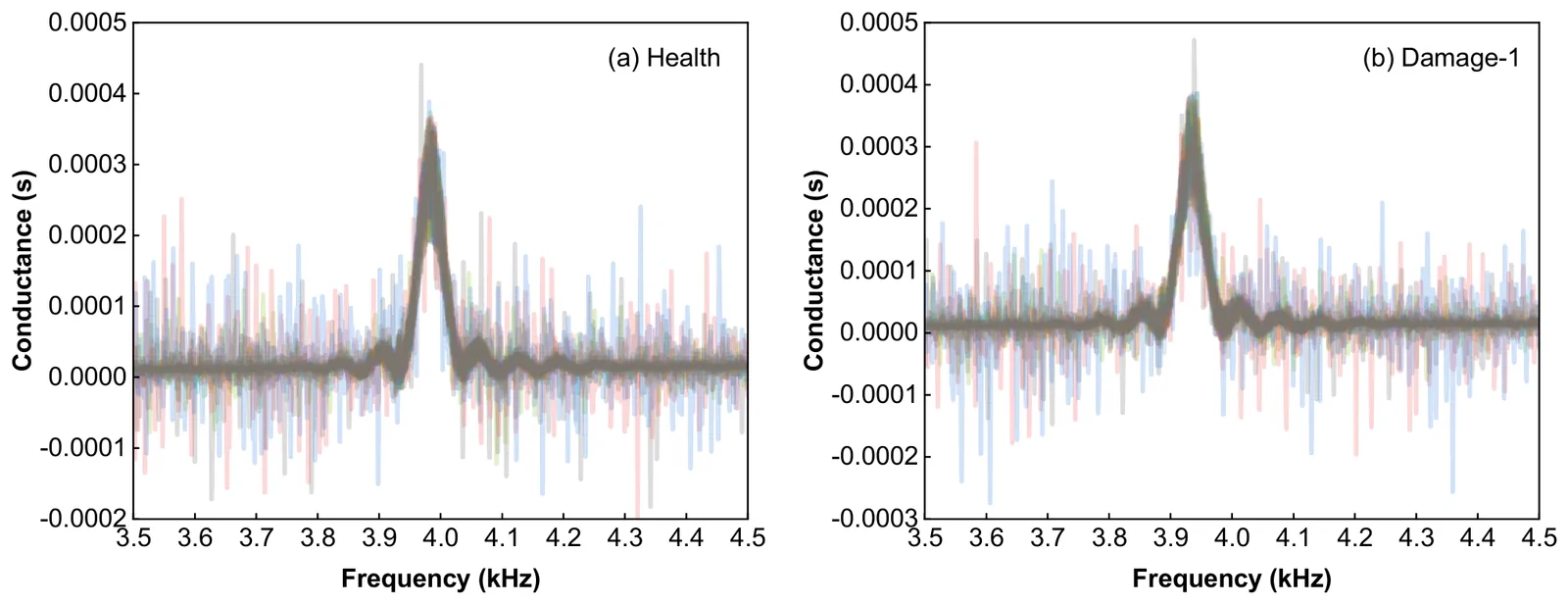

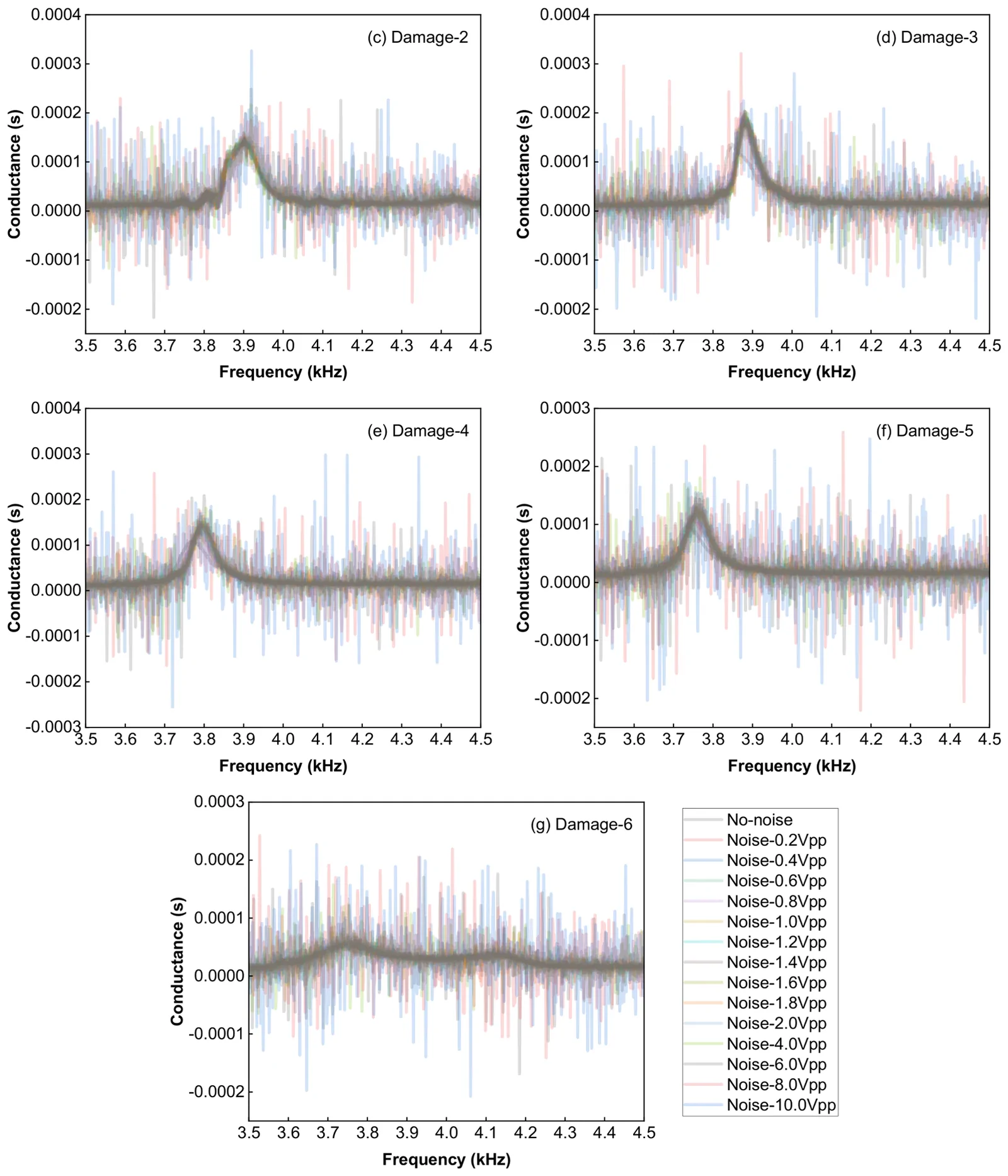
Raw EMI conductance curves for each simulated damage state at different noise levels: (**a**) Health; (**b**) Simulated Damage-1; (**c**) Simulated Damage-2; (**d**) Simulated Damage-3; (**e**) Simulated Damage-4; (**f**) Simulated Damage-5; and (**g**) Simulated Damage-6.

**Figure 8:**
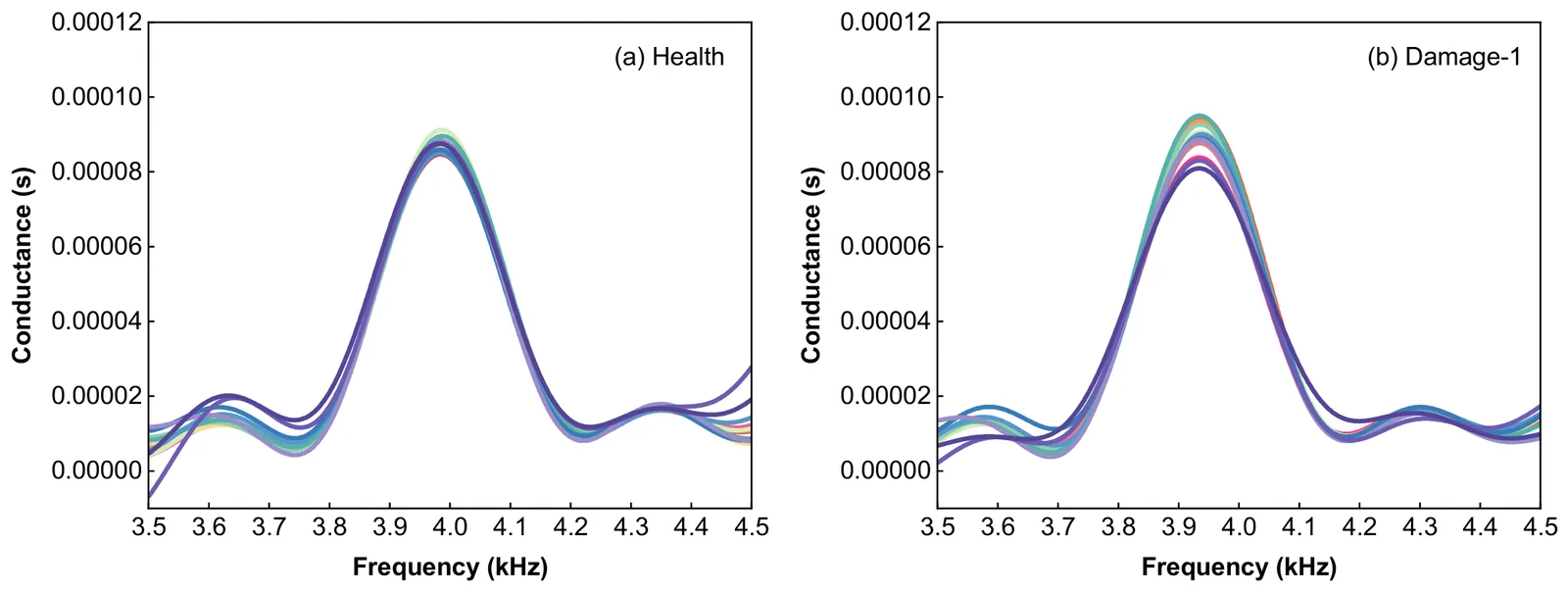

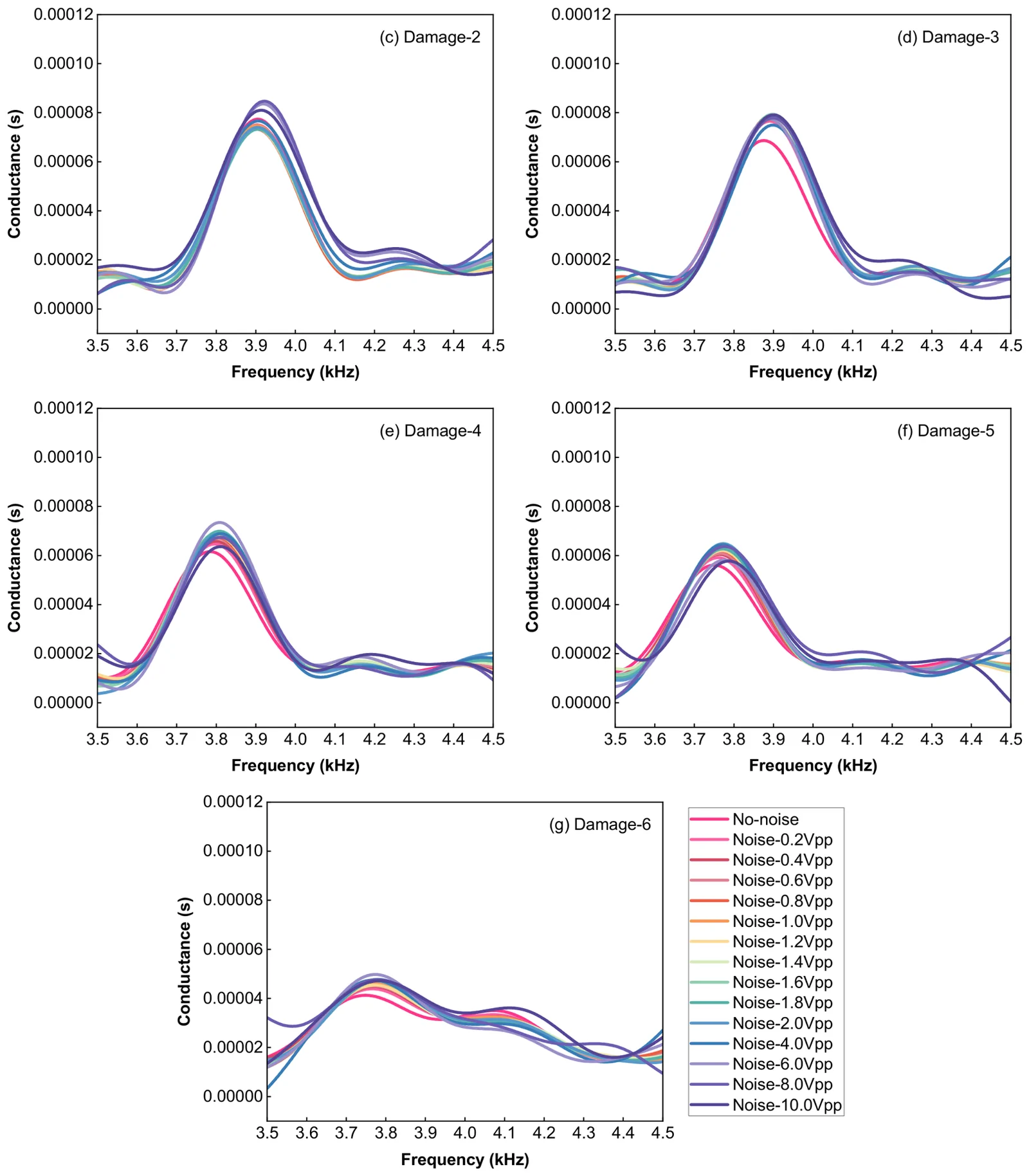
Denoising EMI conductance curves for each simulated damage state at different noise levels: (**a**) Health; (**b**) Simulated Damage-1; (**c**) Simulated Damage-2; (**d**) Simulated Damage-3; (**e**) Simulated Damage-4; (**f**) Simulated Damage-5; and (**g**) Simulated Damage-6.

Figure 7 and Figure 8 show the EMI conductance curves of 105 groups before and after denoising. As shown in Figure 7, the noise signal strongly affects the characterization of damage so that the specific type of damage cannot be distinguished. The overlap of the conductance curves at different noise levels is shown in Figure 7, which reveals that the overlapping black shaded parts clearly shift for different damage types. This explains the use of FFT-SF denoising in this study, and the results of the conductance curve after denoising are shown in Figure 8.

**Figure 9:**
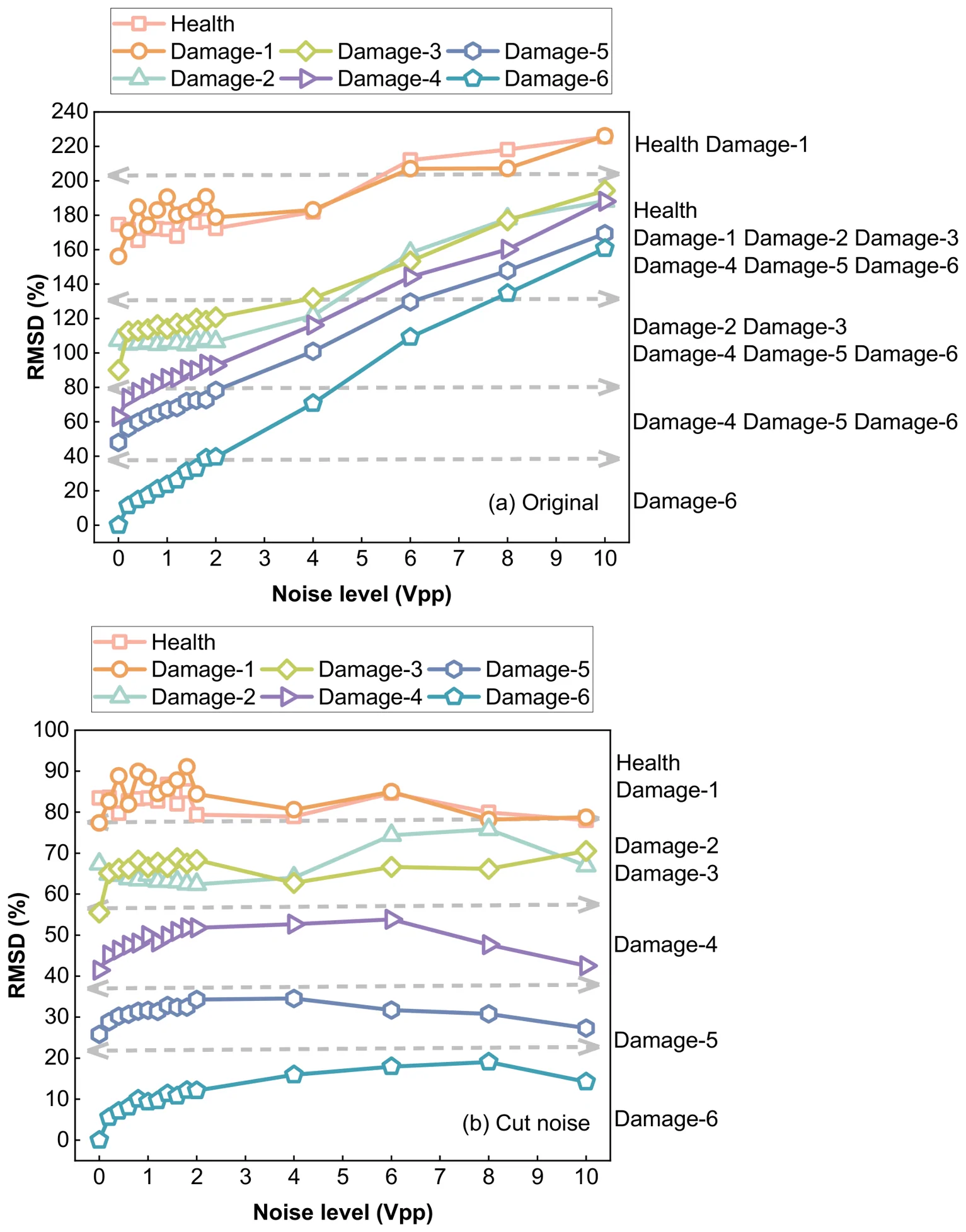
Change in the RMSD indicator with noise level for different simulated damage states: (**a**) before denoising and (**b**) after denoising.

To further quantitatively characterize the degree of damage, the RMSD indicator was used to establish a quantitative relationship under the influence of different damage types and different noise levels. 9a. The 105 sets of data measured were characterized by (Simulated Damage-6, 0.0 Vpp) as the baseline. For each damage type, the RMSD increases with increasing noise level. The determination of noise levels is effective for a single type of damage, but identifying specific types of damage is very inaccurate. This is extremely inconsistent with the simulated damage identification requirements in the actual noisy environment. As shown in Figure 9a, false positives do not occur only when the RMSD is less than 40%, and only Simulated Damage-6 can be detected. When the RMSD is above 40%, the indicator is completely invalid. Therefore, the RMSD constructed by the original conductance curve before denoising alone cannot achieve quantitative identification.

The RMSD results after FFT-SF processing are shown in Figure 9b, which reveals that Simulated Damage-4, Simulated Damage-5, and Simulated Damage-6 are effectively quantified. Moreover, the RMSD of each damage type is suppressed when the noise level changes and tends to flatten or form a clear separator with other damage types. However, there are false positives among Health, Simulated Damage-1, Simulated Damage-2, and Simulated Damage-3, which is not the failure of the FFT-SF denoising method but is caused by the limitations of the RMSD index. According to Ma et al. (Our previous studies) [12,25], the RMSD reflects the difference in the conductance amplitude (longitudinal) across the frequency band and is not sensitive to the frequency offset (lateral) of each point on the spectrum. As shown in Figure 8, the resonant amplitude of Health and Simulated Damage-1 is approximately 0.00009 s, the amplitude of Simulated Damage-2 and Simulated Damage-3 is approximately 0.00008 s, and the difference between their conductance amplitudes is very small, which explains the limitation of the RMSD. Therefore, a more appropriate and accurate quantification method is needed. Moreover, from the limitations of the RMSD index, not considering the physical parameter of the resonant peak offset fully is extremely unfavorable for damage discrimination, which leads to the loss of key damage characteristics in the quantitative model.

### 5.2. Hybrid Physical Features and Data-Driven Quantitative Identification and Prediction of Damage

**Figure 10:**
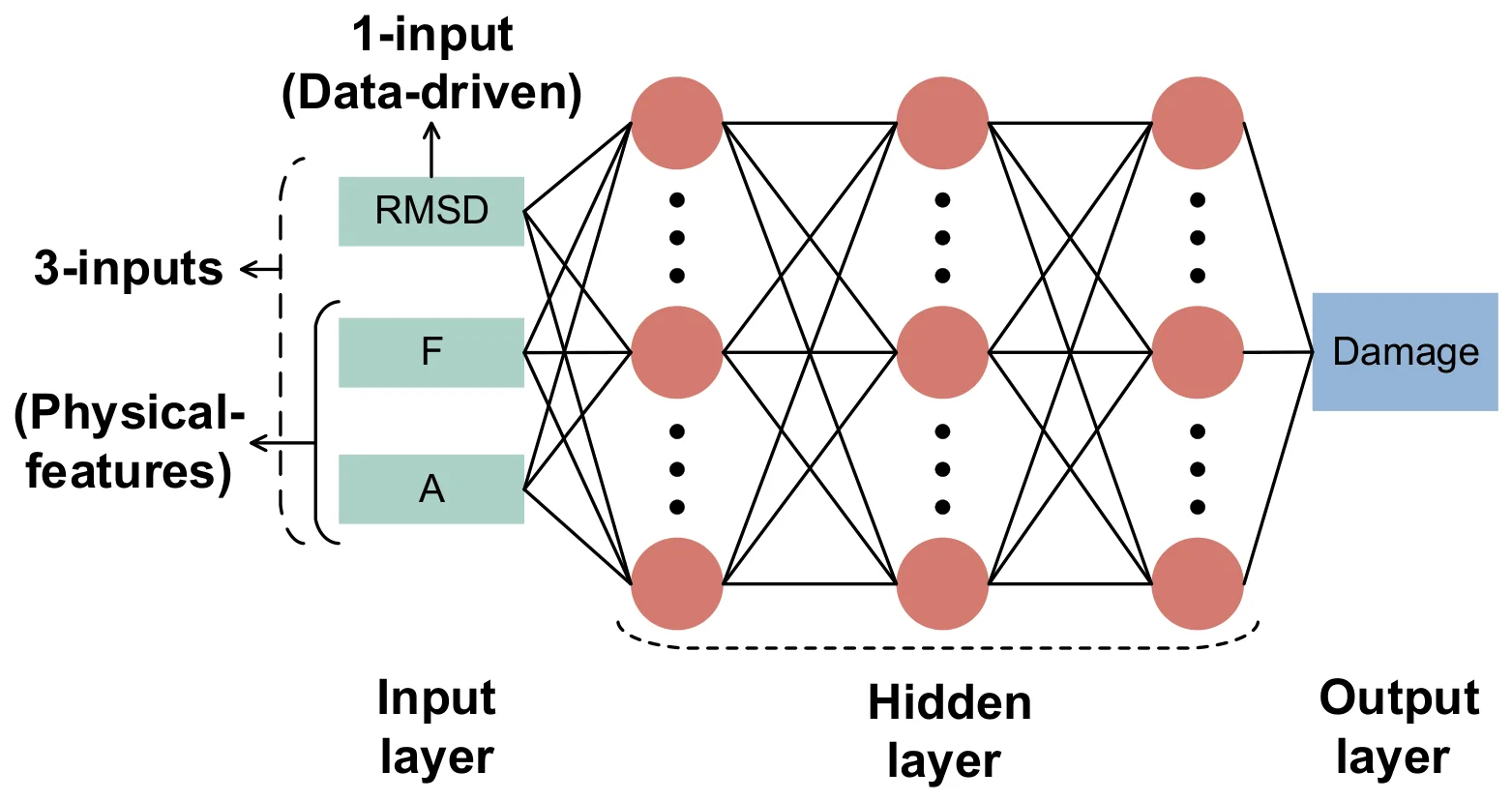
Architecture of the ANN models for predicting damage.

In Section 5.1, this study establishes a traditional quantitative relationship between RMSD indicators and damage types. To further increase the accuracy of quantitative monitoring and achieve the purpose of prediction, in this study, a data-driven method was used to train a data-driven damage prediction model of CFRP beams in a noisy environment with damage type being the target. The original training data of the model are derived from 105 sets of EMI conductance data (denoised by the FFT-SF method); see Figure 8. Two methods for building a training dataset are discussed. The first method is a pure data-driven model that uses only the denoised RMSD indicator as a single input. This was shown to be an effective method in the study of Meher and Sunny [19]. In the previous section, they highlight the necessity of the resonant peak biparameter as a valid physical feature for damage monitoring. Therefore, the second method is a method combining physical features and data-driven model that uses the denoising RMSD index and the resonant peak as the three feature inputs, as shown in Figure 10. The effect of physical features on the model is discussed in Section 5.2.3.

#### 5.2.1. Model Development and Training

In this study, three types of data-driven regression models, XGBoost (XGB), random forest (RF), and a feedforward artificial neural network (ANN), are compared to identify the most suitable architecture for damage prediction. All three are standard supervised learning models that learn a mapping from input features to a continuous damage index through empirical risk minimization. To ensure a fair comparison, the hyperparameters of each model were optimized using the Optuna framework. The optimized hyperparameter configurations for the XGBoost, RF, and ANN models are summarized in Appendix A Table A1, Table A2, Table A3 and Table A4, respectively. Model performance was evaluated using the root mean square error (RMSE) and mean absolute error (MAE).

**Figure 11:**
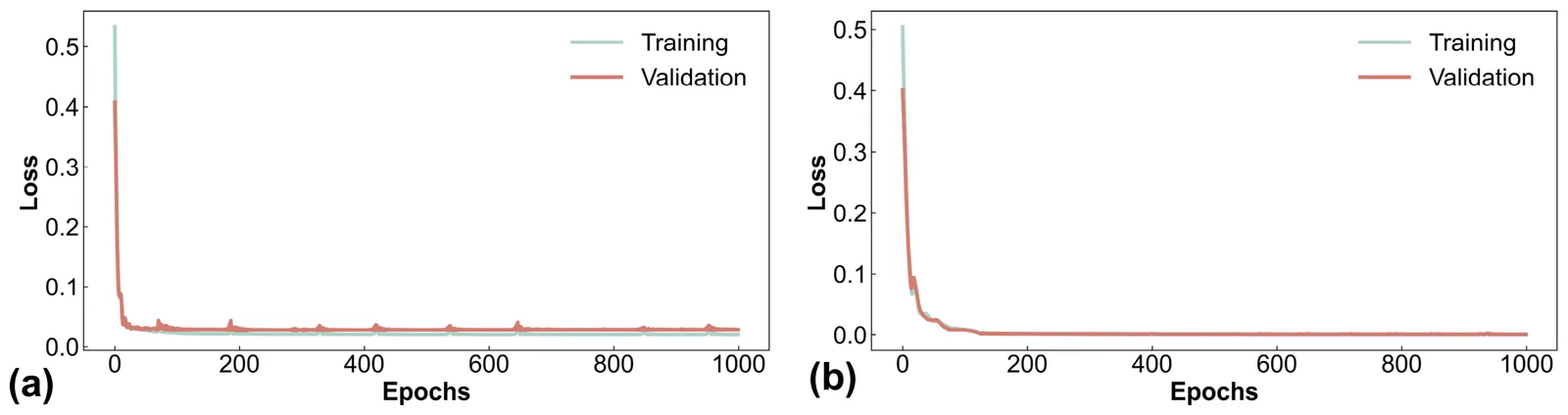
Evolution of the total loss during the training and validation phases for the ANN models: (**a**) 1-input ANN model; (**b**) 3-input ANN model.

The ANN contains three hidden layers, each of which has an ReLU activation function (see Figure 10). Among the 105 sets of data used for model training, each set of data contains 1 or 3 inputs and 1 output: damage (0 represents healthy and 1–6 represents damaged in the output data). A regression formulation was adopted over multi-class classification because the simulated damage states represent progressively increasing structural perturbation with a natural ordering. Regression captures the ordinal nature of these states and provides continuous predictions that reflect the proximity to adjacent states, a capability that is valuable for trend monitoring and early warning applications where the graded severity of damage is as important as the discrete state assignment. For discrete state identification, the continuous prediction ŷ is rounded to the nearest integer within [0, 6]: predicted state = round(ŷ). To prevent data leakage between training and test sets, the splitting was performed at the level of individual noise-level measurements within each simulated damage state, following a stratified sampling strategy. Specifically, for each of the seven simulated damage states, the 15 noise-level replicates were randomly partitioned into training (70%, ≈10 samples), validation (15%, ≈3 samples), and test (15%, ≈2 samples) subsets. This procedure ensures that: (a) each subset contains measurements from all seven states with proportional representation; (b) the test set consists of noise-level measurements not seen during training, thereby evaluating the model’s ability to predict the correct state under novel noise conditions; and (c) no measurement from the same noise-level condition appears in more than one subset. Before training, the input data are preprocessed using RobustScaler normalization. During the training process, the learning rate of the Adam optimizer is 0.01/0.004 (1 input/3 inputs), the maximum number of iterations is 1000, and the loss function of the task is the mean squared error (MSE). The evolution of the training and validation total loss over time is shown in Figure 11. Both the training and validation losses decrease rapidly during the initial epochs, gradually converge, and then stabilize after an inflection point is reached.

#### 5.2.2. Model Performance and Generalization

To evaluate the predictive accuracy and generalization ability of the model, three performance indicators are used: the root mean square error (RMSE), mean absolute error (MAE), and coefficient of determination (R^2^). The formulas for these indicators are as follows:

(9) $$\mathrm{RMSE}=\sqrt{\frac{1}{n}\sum_{i=1}^{n}{\left(y_{i}-\overset{\wedge }{y_{i}}\right)}^{2}}$$

(10) $$\mathrm{MAE}=\frac{1}{n}\sum_{i=1}^{n}\left|y_{i}-\overset{\wedge }{y_{i}}\right|$$

(11) $$R^{2}=1-\frac{\sum_{i=1}^{n}{\left(y_{i}-\overset{\wedge }{y_{i}}\right)}^{2}}{\sum_{i=1}^{n}{\left(y_{i}-\bar{y}\right)}^{2}}$$

In these equations, *y_i_* represents the true value of the *i*-th output, *ŷᵢ* is the corresponding predicted value, *n* is the total number of data points, and *ȳ* represents the average of the true values.

**Figure 12:**
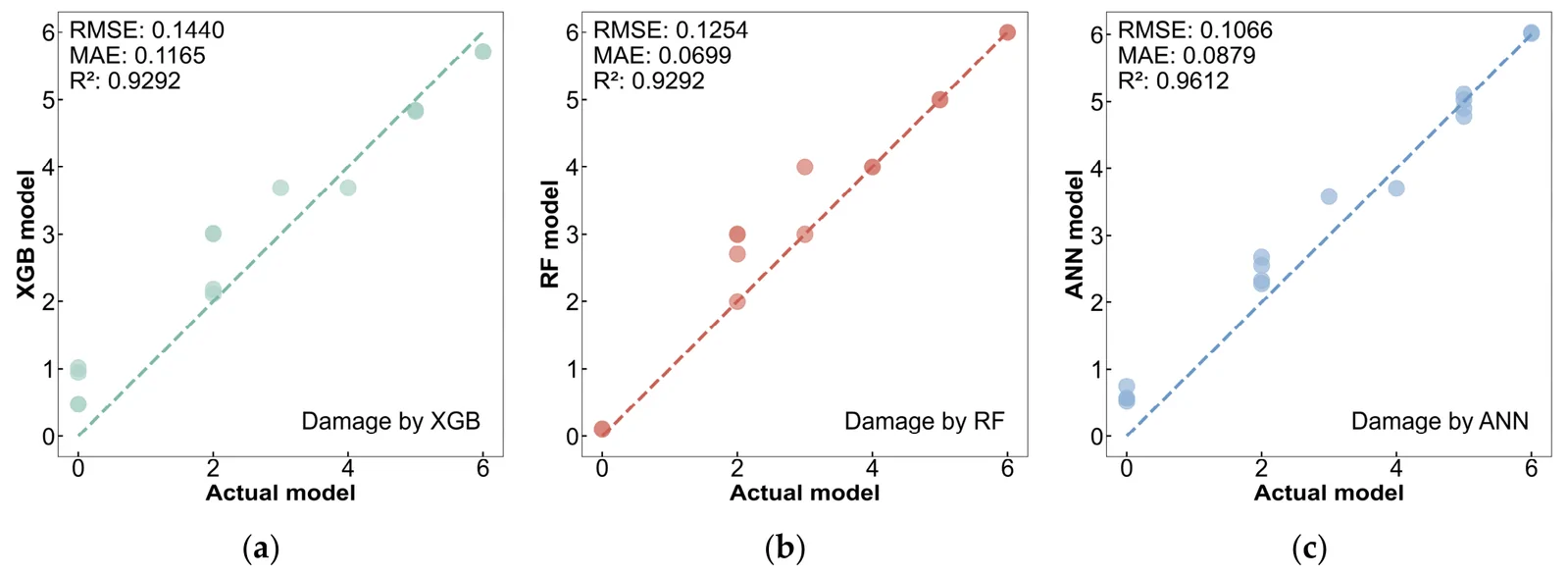
Performances of the 1-input models in predicting damage: (**a**) XGB, (**b**) RF, and (**c**) ANN.

The performance evaluation indicators of the XGB, RF, and ANN models are shown in Figure 12. The horizontal axis represents the experimental true value, and the vertical axis represents the predicted value of the corresponding model. The relevant performance evaluation indicators are shown in Table 3. The experimental results reveal that in the pure data-driven mode, the single-input ANN model based on RMSD features achieves the best comprehensive performance in terms of damage prediction. Specifically, the lowest root mean square error (RMSE = 0.1066) and the highest coefficient of determination (R^2^ = 0.9612) were obtained for damage prediction. With their flexible nonlinear mapping capabilities, ANN models can more effectively mine complex associations in data, resulting in better prediction effects. This addresses the limitation in that RMSD metrics of Health, Simulated Damage-1, Simulated Damage-2, and Simulated Damage-3 cannot be decoupled.

**Figure 13:**
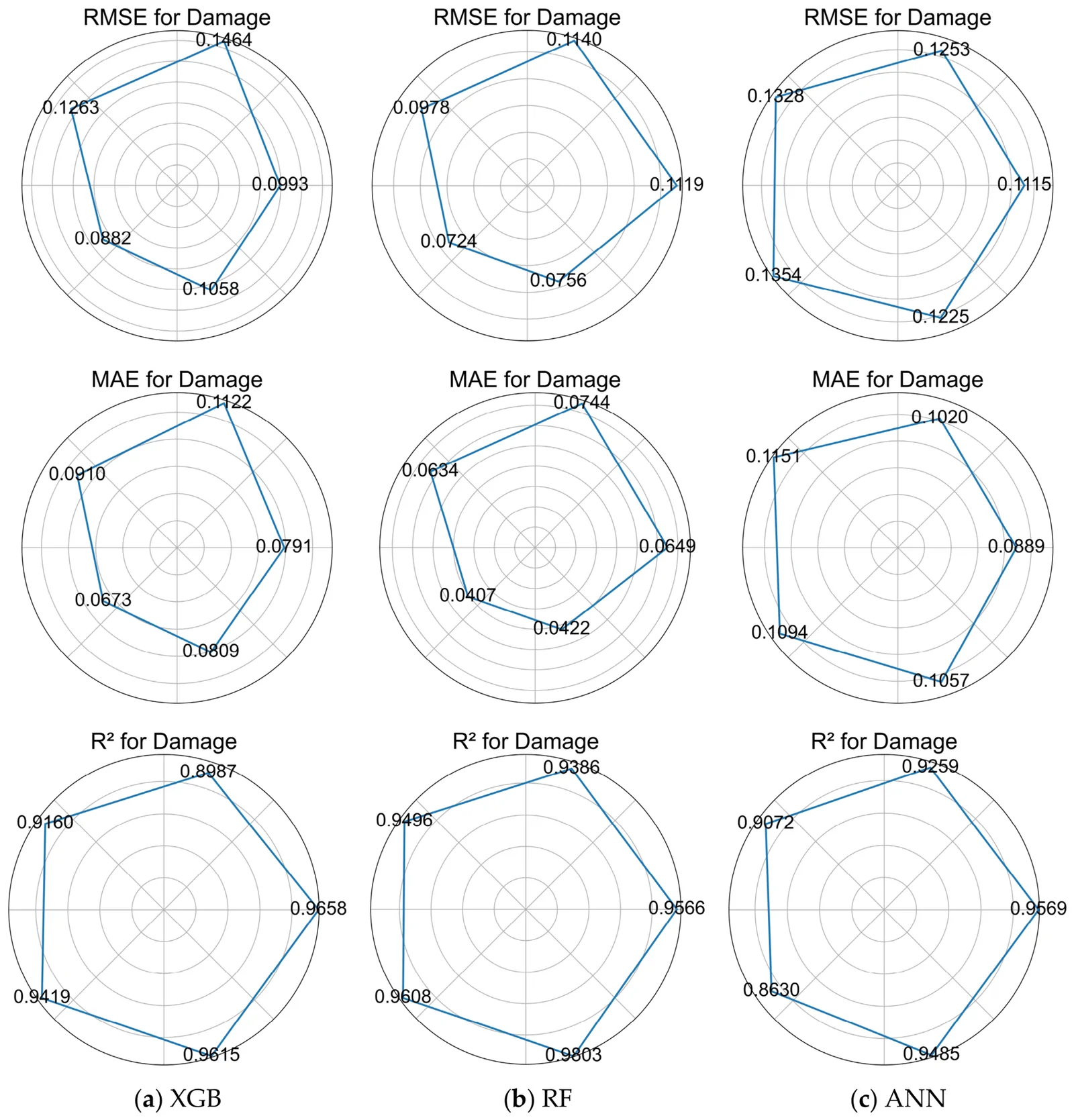
Fivefold cross-validation performance metrics for the XGB, RF, and ANN models (1-input configuration) in predicting damage.

To evaluate the generalization performance of the model on unknown datasets, the performance of the three types of data-driven models is cross-verified by five-fold cross-validation. The training set is randomly divided into five equal subsets; one set is used as a validation set, and the other four sets are used for training. The model’s performance (RMSE, MAE, and R^2^) on each subset is evident in the radar diagram in Figure 13. Although the XGB and RF models outperformed the ANN on several of these subsets, they performed unevenly across all the subsets, indicating that the model was at risk of overfitting [39]. Compared with those of the XGB and RF models, the performance evaluation metrics of the ANN model are more uniform across all the subsets (closer to the regular pentagon).

These findings indicate that the ANN model can reliably identify the correct simulated damage state under noise conditions not seen during training, demonstrating robust generalization across noise levels. We emphasize that the primary objective of the cross-validation strategy employed here is to assess the model’s ability to recognize known structural states when presented with novel noise interference, a scenario directly relevant to practical SHM, where the structure may be in a previously characterized condition but measurements are acquired under varying, unpredictable environmental noise. The 5-fold random-split design serves this objective by ensuring that the test set contains noise-level measurements excluded from training, thereby evaluating noise-robust recognition performance.

It should be noted that this validation strategy does not assess the model’s ability to extrapolate to entirely unseen structural states. A leave-one-state-out cross-validation, where the model is trained on six states and tested on the held-out seventh state, would evaluate this different capability. Given that the seven magnet-induced states represent a progressive perturbation with smoothly varying EMI signatures, the primary practical challenge in our experimental context is not inter-state extrapolation but rather within-state recognition under varying noise, precisely the capability that the current validation demonstrates. This distinction between noise generalization and state extrapolation is explicitly acknowledged, and the latter is identified as a direction for future work in Section 6.

#### 5.2.3. Discussion: Enhancing the Model Capability Through Dual Features of the Resonance Peak

**Figure 14:**
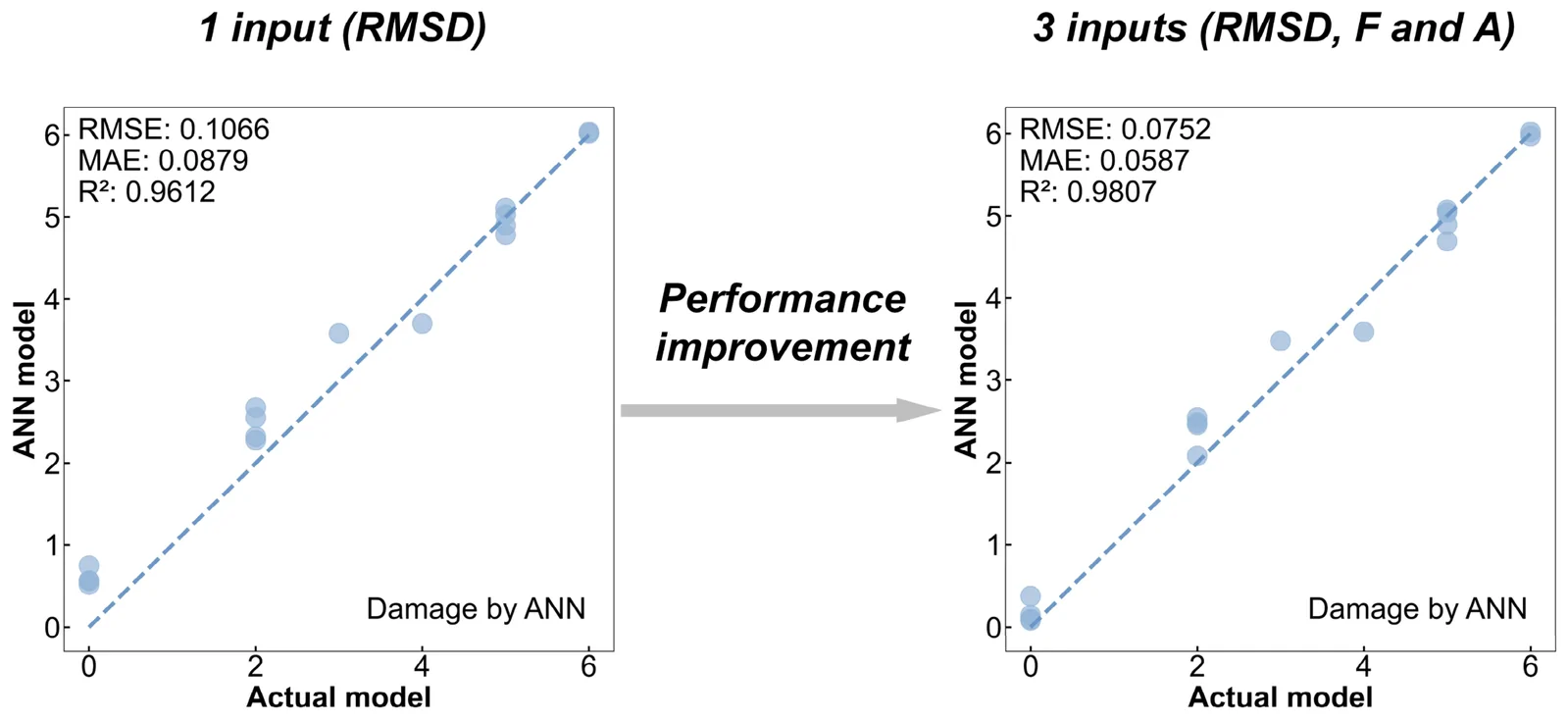
Performance of the 1-input and 3-input ANN models in predicting damage.

In the aforementioned study, the dual parameter of the resonant form is the key physical feature in terms of the performance of the main structure. Combining these two features with the RMSD, the ANN model with better performance is selected to train the method combining physical features and the data-driven model regarding the input of the three features. Moreover, hyperparameter optimization is performed again (see Table A4 in Appendix A), and the model performance is improved, as shown in Figure 14. Compared with the pure data-driven ANN model that uses only RMSD input, the introduction of two additional physical features yields a consistent improvement in prediction accuracy across all evaluation metrics, as shown by the lower RMSE, higher R^2^, and more stable cross-validation performance. While formal statistical significance testing is constrained by the limited number of cross-validation folds, the reproducibility of this improvement across multiple independent evaluation dimensions, including the five-fold cross-validation, the full test set metrics, and the SHAP analysis, supports the conclusion that the physical features F and A provide meaningful additional discriminative information. Moreover, the quality and quantity of physical features are crucial to model performance, and effective features can significantly improve the prediction ability.

**Figure 15:**
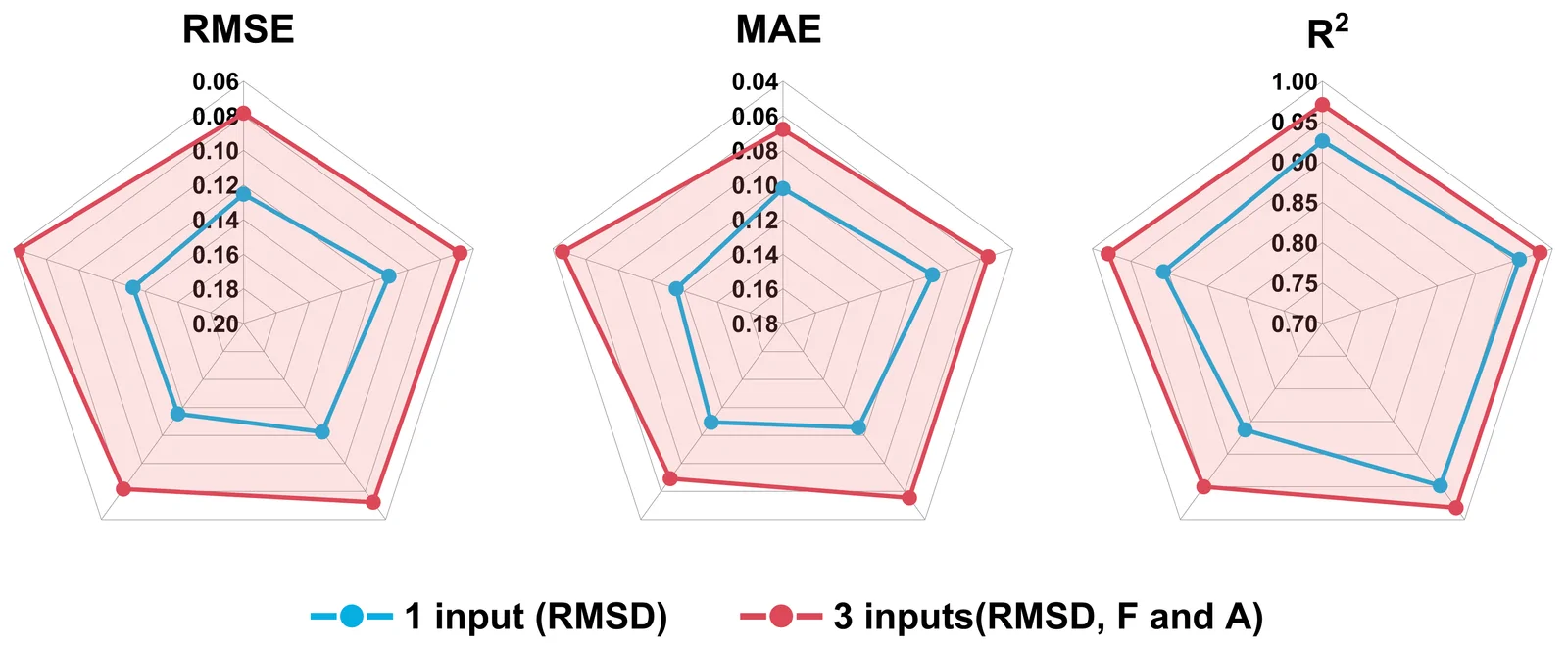
Five-fold cross-validation performance metrics for the ANN model (1-input and 3-input configurations).

The 3-input ANN model achieves strong performance on the test set (RMSE = 0.0752, MAE = 0.0587, R^2^ = 0.9807), representing a meaningful improvement over the 1-input baseline. A five-fold cross-validation test was also performed to check whether the model had a risk of fitting, as shown in Figure 15. We note that the five-fold cross-validation results confirm the stability of this improvement across different data partitions. The 3-input ANN model not only avoids overfitting but also shows better generalization performance than the 1-input ANN model. The performance of the 1-input ANN model was limited by the small dataset of 105 samples, a common challenge for purely data-driven models that typically require large training sets to achieve optimal performance.

To further evaluate the classification performance, the continuous ANN output was converted to a discrete state by rounding to the nearest integer within [0, 6]. For example, a predicted value of 0.101 for the Health state (true label 0) yields a rounded value of 0, corresponding to correct classification. Table 4 presents representative test cases that illustrate the comparative performance of the 1-input and 3-input ANN models. For each case, the accuracy was computed from the continuous prediction as (1 − |ŷ − y_true|/6) × 100%, reflecting the proximity of the prediction to the true state. In all cases, the 3-input model achieves substantially higher accuracy, particularly for Health and Simulated Damage-1 where the RMSD values alone are insufficient for reliable discrimination.

**Figure 16:**
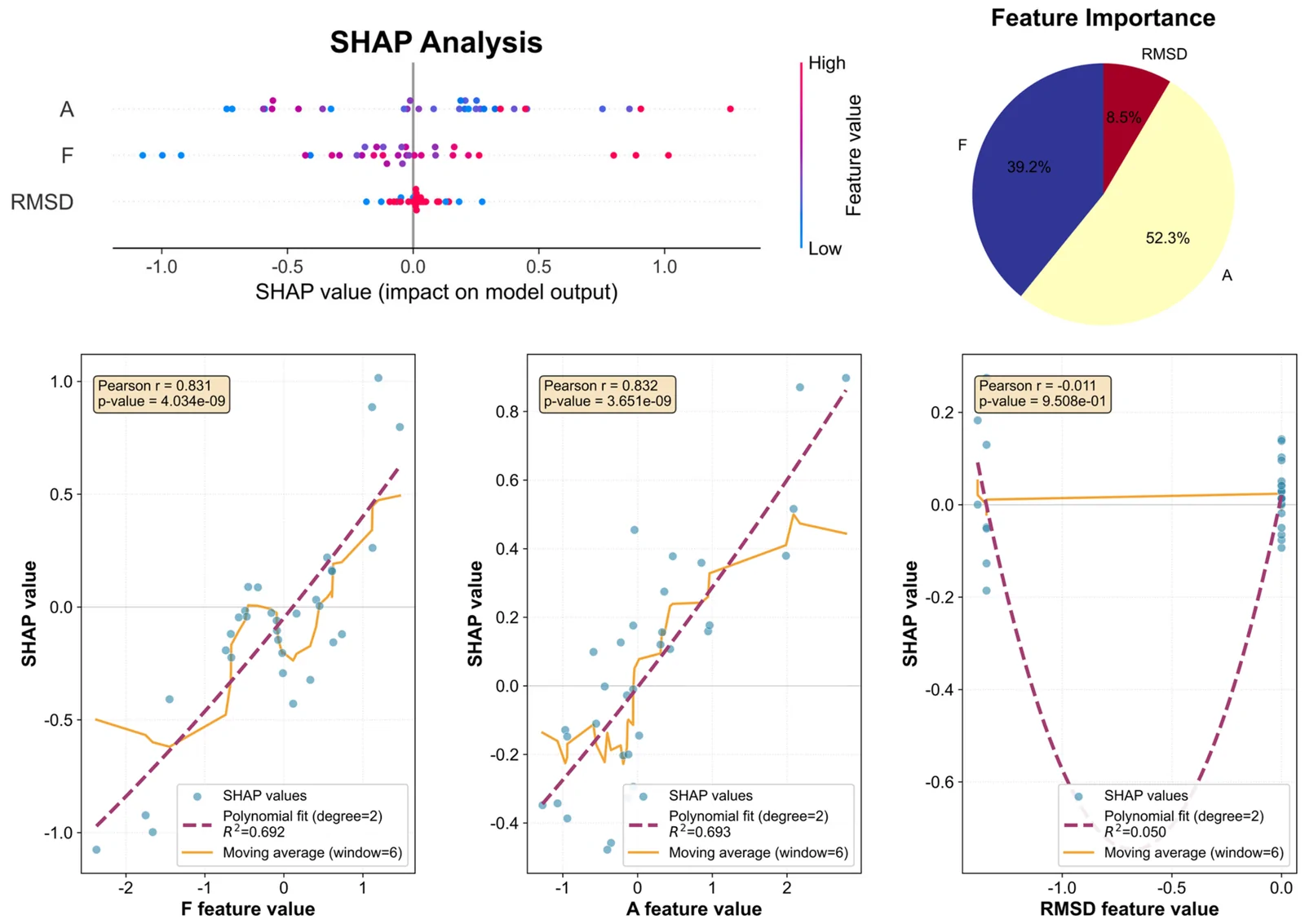
SHAP analysis, feature importance, and dependence plots of the best model (3-input ANN).

In the above case, the RMSD, as a single-input, data-driven model, reflects a large deviation between the predicted value and the actual value, which means that the reliability of its predictive results is low. As a statistical quantitative index of the entire EMI spectrum, the RMSD is easy to calculate, but the physical features related to the change in the main structure state are lost during the calculation. In contrast, the additional resonant dual parameters introduced by the 3-input ANN model more comprehensively characterize the quantization relationship between the input and output and ultimately yield higher prediction accuracy and generalizability. The SHAP analysis was performed using KernelSHAP with background samples randomly drawn from the training set. The pairwise Pearson correlation coefficients among the three input features were r(RMSD, F) = −0.62, r(RMSD, A) = −0.71, and r(F, A) = 0.58. These moderate correlations are physically expected: as simulated damage progresses, stiffness decreases (F decreases), damping increases (A decreases), and the spectral deviation from the baseline increases (RMSD increases), reflecting coupled but distinct aspects of the structural state change. Figure 16 presents the Shapley Additive Explanation (SHAP) analysis of the physical features. The feature importance plot reveals that the EMI dual features of the resonance peak contribute substantially more to the model predictions than the RMSD index, underscoring the critical role of physical features in enhancing prediction accuracy. The dependence plots reveal how these features influence predictions across their measured ranges: as F decreases, indicating stiffness reduction, the SHAP value becomes increasingly negative, driving predictions toward higher simulated damage states, while decreasing A, indicating increased damping, produces a similar trend. Figure 16 further reveals an interaction effect between F and A, where stiffness loss and increased damping act synergistically on the model output. This pattern is physically consistent with the electromechanical coupling principles described in Section Physical Interpretation of the Resonance Features, confirming that the ANN has learned physically meaningful relationships from the data.

A direct quantitative comparison of the proposed framework with prior EMI-based methods on an identical dataset is constrained by several factors. Existing studies employed different structural materials and geometries, sensor configurations, damage simulation approaches, and noise injection protocols. These experimental differences fundamentally affect the baseline EMI signatures and noise characteristics, precluding a fair like-for-like numerical comparison of metrics such as RMSE or classification accuracy. Furthermore, several prior studies did not report the full set of performance metrics required for comprehensive benchmarking. The qualitative advantages of the proposed framework relative to existing approaches can nonetheless be articulated. Compared with index-stabilization methods, FFT-SF uniquely reconstructs the resonance signature rather than merely stabilizing the damage index, enabling physics-based feature extraction. Compared with EMI-ML methods, the proposed framework operates with substantially fewer training samples and without synthetic data augmentation. The SHAP analysis further provides interpretability that black-box deep learning models lack. These qualitative distinctions, supported by the internal quantitative evidence within this study (DC = 87.8%, R^2^ = 0.9807 on 105 samples, SHAP feature importance), collectively demonstrate the contribution of the proposed framework.

The trends observed in this study, monotonic leftward frequency shift and amplitude attenuation with increasing magnet loading, are qualitatively consistent with the expected effects of stiffness-reducing and damping-increasing damage mechanisms. Delamination reduces local bending stiffness by decoupling adjacent plies, which would similarly produce a leftward shift in F, while matrix cracking increases internal friction and energy dissipation, which would manifest as reduced A. The RMSD, as a broadband statistical measure, captures the global spectral deviation resulting from both effects. These mechanistic analogies suggest that F, A, and RMSD are likely to remain informative features under real damage conditions, as they directly reflect the stiffness and damping changes that are common to many degradation modes.

However, several important differences between magnet loading and real damage must be considered when assessing the quantitative transferability of the present results. First, real delamination involves nonlinear contact dynamics at the delaminated interfaces, including opening and closing under vibration, that may produce amplitude-dependent resonance shifts not captured by the linear magnet-induced perturbation. This could result in F shifts that vary with excitation level or damage severity in ways not observed in this study. Second, distributed matrix cracking affects a larger volume of material and may influence multiple resonance modes simultaneously, whereas magnet loading produces a localized perturbation that primarily affects a single dominant mode. Third, fiber breakage introduces abrupt, irreversible stiffness discontinuities that may produce qualitatively different spectral features, such as the appearance of new resonance peaks or mode splitting, rather than the smooth, monotonic trends observed under progressive magnet loading. These differences imply that while the qualitative trends (frequency decrease, amplitude attenuation) are expected to persist, the quantitative mapping from feature values to specific damage states learned by the ANN on magnet-simulated data would require recalibration, and possibly architectural modification, when applied to real damage scenarios. The core methodology, however, FFT-SF spectral reconstruction followed by physics-based feature extraction and ANN-based state mapping, remains agnostic to the perturbation mechanism and should remain applicable provided that the induced EMI signature changes are measurable and distinguishable across damage states. Systematic validation on specimens with controlled real damage remains the essential step for quantifying the framework’s performance under realistic conditions.

To evaluate data efficiency, the 3-input ANN was trained on progressively reduced subsets of the full dataset: 100% (105 samples), 75% (79 samples), 50% (53 samples), and 25% (26 samples), with stratified sampling to maintain proportional state representation. The average RMSE and R^2^ are reported in Appendix A Table A6. Performance degrades substantially as data are reduced: RMSE increases from 0.0752 (100% data) to 0.2715 (25% data), while R^2^ decreases from 0.9807 to 0.7634. The sharpest decline occurs between 50% and 25% data, where each simulated damage state is represented by only 3–4 samples, providing insufficient coverage of the noise-level variation within each state.

In this study, the PZT sensor was used to directly extract high-quality feature data (including 1 data-driven feature and 2 physical features) and acted as a front-end feature extractor [40]. This approach of integrating the extracted resonance frequency and amplitude features into an ANN was a physical features machine learning method [41]. Unlike other studies that use deep learning to extract implicit features from sensor raw data [18], this method captures the main physical features of the sensor and the main electromechanically coupled structure, effectively characterizing the changes in CFRP beams in the simulated damage state. This strategy clarifies the correlation between the basic characteristics of the sensor and the mechanical properties of the main structure, and the key features of the EMI signal have better physical interpretability potential (as shown in Figure 16) than the “black box” features and RMSD learned from the original data by the deep learning model. At present, the key limitation of EMI technology is the extraction of decisive physical features for health diagnosis from the EMI spectrum, and this study demonstrates the great potential of this concept by using a combination of physical features and data-driven framework for CFRP beams simulated damage identification prediction. This idea can be applied to various health diagnosis problems, and it can save many computing resources compared with black-box deep learning methods.

It is important to clarify that the term “physical features” in this work refers to physically meaningful quantities (resonance frequency and amplitude) extracted through domain knowledge, rather than the embedding of governing physical equations as constraints within the learning algorithm (as in physics-informed neural networks, PINNs). This feature engineering approach leverages the well-established electromechanical coupling principles of EMI technology to provide the ANN with inputs that have direct physical interpretations.

## 6. Conclusions

In this study, a framework combining electromechanical impedance (EMI) technology, an EMI-specific frequency-domain adaptation of the fast Fourier transform smoothing filter (FFT-SF), and an artificial neural network (ANN) is developed and experimentally evaluated. The core methodological contribution lies in the integrated combination of FFT-SF spectral reconstruction, which enables reliable extraction of resonance features from noise-corrupted EMI spectra, with dual physical feature extraction and ANN-based state mapping, tailored to address the specific challenge of noise-robust, small-sample simulated damage identification in EMI-based SHM. Through this integrated framework, accurate identification and prediction of simulated damage states in a CFRP composite beam under random noise interference are achieved. The main conclusions are as follows:

(1) In this study, the influence of the noise environment on simulated damage identification is successfully eliminated by introducing an EMI-based FFT-SF denoising method. Combined with the results of the analysis of the RMSD quantitative indices, the efficiency of FFT-SF is improved compared to that of the previous denoising technology. FFT-SF is used to process conductance signals, which overcomes the limitations of traditional denoising technology and extracts key physical feature information of resonant peaks. Under random noise interference, the RMSD treated by FFT-SF can accurately identify simulated damage states 4–6. However, owing to the limitations of the RMSD, some simulated damage states cannot be quantitatively decoupled.
(2) Based on the RMSD characteristics, three pure data-driven models are constructed and optimized for the quantitative identification and prediction of damage under the influence of random noise. Comparative analysis revealed that the artificial neural network (ANN) model is significantly better than the XGBoost and random forest models in terms of predictive accuracy (RMSE and R^2^) and generalizability and can effectively mine complex nonlinear associations from monitoring data.
(3) In this study, the single-input ANN model with only RMSD features is further compared with the three-input ANN model with physical features (including the RMSD and dual parameters F and A for the resonant peak). The SHAP analysis shows that the introduction of additional dual resonant peak parameters greatly increases the prediction accuracy and generalization performance of the three-input ANN model and addresses the limitations of RMSD indicators in damage decoupling recognition.

A clear and interpretable connection between the electromechanical coupling characteristics of the sensor and the main structure is established by the key dual resonant features captured from the denoising conductance signals. Compared with the pure data-driven model, the introduction of physical features can achieve high-precision modelling under small-sample conditions. Moreover, compared with the black-box features extracted by the deep learning model, these key sensing features improve the physical interpretability of the ANN model.

This study proposes a methodology for simulated damage identification in composite beams; however, several important limitations must be acknowledged. The reported performance metrics were obtained from measurements on a single CFRP beam specimen with magnet-induced structural state changes. Consequently, these results reflect the model’s ability to generalize across noise conditions for known structural states on this specific specimen, rather than demonstrating generalization to independent specimens with manufacturing variability or to structures with different geometries. Cross-specimen validation and extension to complex geometries remain necessary steps before the framework can be considered validated for practical deployment. First, the magnet-based damage simulation employed in this study, which induces local mass and stiffness variations through magnetic attraction, provides a controlled and repeatable means of generating progressive “damage” states for methodological validation. However, this approach primarily simulates reversible local property changes rather than the irreversible microstructural damage mechanisms typical of composite materials, such as delamination, matrix cracking, and fiber breakage. Actual damage modes in CFRP structures involve complex energy dissipation mechanisms (e.g., matrix-fiber debonding, ply separation) that may produce different electromechanical impedance signatures from those observed in this study. Therefore, comprehensive validation on CFRP specimens with authentic impact-induced or fatigue-induced damage is necessary to confirm the transferability of the proposed framework to real-world applications. This validation will be a priority in our future investigations. Second, conductance signatures exhibit sensitivity to temperature variations, necessitating the incorporation of environmental temperature effects in future work. Third, the noise model employed herein represents a simplified approximation, whereas operational environments involve complex noise sources such as structural vibrations and engine harmonics. Finally, the current validation is limited to CFRP beams and focuses on generalization across noise conditions rather than extrapolation to unseen damage types; the latter, along with extension to complex geometries and full-scale structures, will be explored in future work.

## Figures and Tables

**Table 1:** Properties of fibers and epoxy used in composite production.

Material	Density (g/cm^3^)	Tensile Strength (MPa)	Strain at Fracture (%)	Elastic Modulus (GPa)	Manufacturer
Carbon Fiber Fabric	1.79	4500–4900	1.5–2	250	Teijin (Tokyo, Japan)
Epoxy	1.2	50–60	4–6	3.4	Tervan

**Table 2:** Material properties of the PZT sensor.

Property	Value
Density, ρ (kg/m^3^)	7600
Elastic modulus, E (GPa)	67
Poisson’s ratio, ν	0.32
Piezoelectric strain coefficient, $d_{31}$/$d_{32}$/$d_{33}$/$d_{24}$/$d_{15}$(10^−10^ m/V)	−3.6/−3.6/5.0/ 5.8/5.8
Dielectric constant, $\varepsilon_{11}^{\sigma }$/$\varepsilon_{22}^{\sigma }$/$\varepsilon_{33}^{\sigma }$(10^−8^ F/m)	1.75/1.75/2.12

**Table 3:** Performance evaluation metrics of the ANN, RF, and XGBoost models for damage prediction with 1 input feature.

Model	Metric	Damage (1 Input)
	RMSE	0.1066
ANN	R^2^	0.9612
	MAE	0.0879
	RMSE	0.1254
RF	R^2^	0.9292
	MAE	0.0699
	RMSE	0.1440
XGBoost	R^2^	0.9292
	MAE	0.1165

**Table 4:** Comparison of the performance of ANN models on representative test cases.

Model	Actual Damage	Predicted Damage	Damage Accuracy (%)	Average Accuracy (%)
ANN with 3 inputs	0 (Health)	0.101	98.32	98.14
	1	0.827	97.12	
	5	4.938	98.97	
ANN with 1 input	0 (Health)	0.619	89.68	93.83
	1	0.639	93.99	
	5	4.868	97.81	

Note: The continuous ANN prediction ŷ is converted to a discrete state by rounding to the nearest integer: predicted state = round(ŷ). Accuracy = (1 − |ŷ − y_true|/6) × 100%. For the Health case with ŷ = 0.101, round(0.101) = 0, giving correct discrete classification (100% discrete accuracy); the reported 98.32% reflects the small continuous residual.

## Data Availability

The original contributions presented in this study are included in the article. Further inquiries can be directed to the corresponding author.

## Appendix A

**Table A1:** Optimized hyperparameters and search ranges for the XGBoost Model.

Hyperparameter	Optimal Value	Search Range
n_estimators	85	10–200
n_layers	6	3–10
learning_rate	0.1	0.01–0.1
subsample	0.9	0.6–1
colsample_bytree	0.9	0.6–1

**Table A2:** Optimized hyperparameters and search ranges for the RF Model. Author Statement.

Hyperparameter	Optimal Value	Search Range
n_estimators	34	10–100
max_depth	5	3–10
min_samples_split	3	1–10
min_samples_leaf	1	1–10
bootstrap	True	—

**Table A3:** Optimized hyperparameters and search ranges for the 1-input ANN Model.

Hyperparameter	Optimal Value	Search Range
hidden_layer_size	30, 32, 28	10–100
n_layers	3	2–5
activation	ReLU	—
solver	Adam	—
α	0.0001	0.1–0.00001
learning_rate	0.01	—

**Table A4:** Optimized hyperparameters and search ranges for the 3-inputs ANN Model.

Hyperparameter	Optimal Value	Search Range
hidden_layer_size	12, 24, 6	10–100
n_layers	3	2–5
activation	ReLU	—
solver	Adam	—
α	0.01	0.1–0.00001
learning_rate	0.004	—

**Table A5:** Summary of representative studies on EMI-based structural health monitoring.

Reference	Method	Feature Type	Noise Handling	Data Requirement	Interpretability	Key Limitation
De Castro et al. [14]	Hinkley criterion	Statistical indices	Moderate	Small	Low (index only)	False alarms remain; no physics-based features
De Castro et al. [15]	CCSD	Statistical indices	Moderate	Small	Low	No resonance feature extraction
Campeiro et al. [17]	EMI + Lamb waves	Statistical indices	Moderate	Small	Low	Comparative study; no ML integration
Meher & Sunny [19]	RMSD + ANN	Statistical indices	Not addressed	Large	Low (black-box)	Requires extensive training data
Ai & Yang [20]	Transfer learning	Raw admittance features	Not addressed	Large (augmented)	Low	Synthetic data augmentation
Deng et al. [21]	Deep learning (CNN)	Full spectra	Not addressed	Large (augmented)	Low (black-box)	Limited interpretability; high computational cost
Ai & Cheng [18]	2D-CNN	Raw impedance spectra	Not addressed	Large	Low (black-box)	Requires large datasets
This work	FFT-SF + ANN	Physics-based (F, A) + Statistical (RMSD)	High (DC = 87.8%)	Small (105 samples)	High (SHAP analysis)	Magnet-simulated damage; white noise only

**Table A6:** Effect of dataset size on model performance for the 3-input ANN.

Dataset (%)	Samples	RMSE	R^2^
100	105	0.0752	0.9807
75	79	0.1187	0.9483
50	53	0.1843	0.8826
25	26	0.2715	0.7634
